# Phylogenomics and topological conflicts in the tribe Anthospermeae (Rubiaceae)

**DOI:** 10.1002/ece3.10868

**Published:** 2024-01-25

**Authors:** Olle Thureborn, Niklas Wikström, Sylvain G. Razafimandimbison, Catarina Rydin

**Affiliations:** ^1^ Department of Ecology, Environment and Plant Sciences Stockholm University Stockholm Sweden; ^2^ The Bergius Foundation The Royal Academy of Sciences Stockholm Sweden; ^3^ Department of Botany The Swedish Museum of Natural History Stockholm Sweden

**Keywords:** inversions, mitochondrial DNA, mitochondrial DNA insertion, nuclear ribosomal DNA, phylogenetic discordance, plastid DNA

## Abstract

Genome skimming (shallow whole‐genome sequencing) offers time‐ and cost‐efficient production of large amounts of DNA data that can be used to address unsolved evolutionary questions. Here we address phylogenetic relationships and topological incongruence in the tribe Anthospermeae (Rubiaceae), using phylogenomic data from the mitochondrion, the nuclear ribosomal cistron, and the plastome. All three genomic compartments resolve relationships in the Anthospermeae; the tribe is monophyletic and consists of three major subclades. *Carpacoce* Sond. is sister to the remaining clade, which comprises an African subclade and a Pacific subclade. Most results, from all three genomic compartments, are statistically well supported; however, not fully consistent. Intergenomic topological incongruence is most notable in the Pacific subclade but present also in the African subclade. Hybridization and introgression followed by organelle capture may explain these conflicts but other processes, such as incomplete lineage sorting (ILS), can yield similar patterns and cannot be ruled out based on the results. Whereas the null hypothesis of congruence among all sequenced loci in the individual genomes could not be rejected for nuclear and mitochondrial data, it was rejected for plastid data. Phylogenetic analyses of three subsets of plastid loci identified using the hierarchical likelihood ratio test demonstrated statistically supported intragenomic topological incongruence. Given that plastid genes are thought to be fully linked, this result is surprising and may suggest modeling or sampling error. However, biological processes such as biparental inheritance and inter‐plastome recombination have been reported and may be responsible for the observed intragenomic incongruence. Mitochondrial insertions into the plastome are rarely documented in angiosperms. Our results indicate that a mitochondrial insertion event in the plastid *trnS* ^GGA^ – *rps4* IGS region occurred in the common ancestor of the Pacific clade of Anthospermeae. Exclusion/inclusion of this locus in phylogenetic analyses had a strong impact on topological results in the Pacific clade.

## Graphical abstract

Genomic data have provided new opportunities to address phylogenetic incongruence and its bases. Our work shows notable intergenomic incongruence in a tribe of the coffee family, and the null hypothesis of congruence among all sequenced loci in the individual genomes was surprisingly rejected for plastid data. We further detected a (rarely reported) insertion of mitochondrial data into the plastome that had a decisive impact on tree topology.

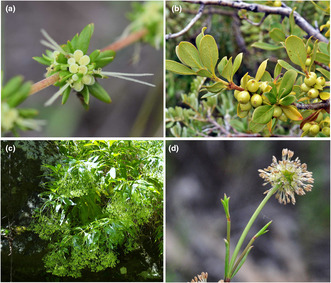

## 1 INTRODUCTION

The recent advent of high‐throughput sequencing (HTS) techniques has allowed researchers to assemble large‐scale genomic DNA datasets (Straub et al., 2012). The immense increase in sequenced base pairs available for phylogenetic studies has provided plant systematists an opportunity to infer more resolved and better supported phylogenetic hypotheses both on deeper and shallower levels. Shallow whole‐genome sequencing, also known as genome‐skimming (Straub et al., 2012), targets the high copy regions of the genome, including the organellar genomes (i.e., the plastome and mitogenome), and the nuclear ribosomal cistron. Typically, low‐coverage sequencing (ca 1×) of the genomic DNA is enough to obtain complete or at least large fractions of the mitogenome, plastome, and the nuclear ribosomal cistron (Berger et al., 2017; Malé et al., 2014; Straub et al., 2012). Thus, DNA sequence data obtained via genome skimming will provide a sufficient basis for comparative phylogenetic studies of results from the three different genomes.

Among the three compartments from which DNA sequence data are extracted using genome skimming, the chloroplast genome has been the most widely used, utilized in studies ranging from intraspecific investigations to Viridiplantae‐wide phylogenetic work (e.g., Gitzendanner et al., 2018; Jiang et al., 2019; Ruhfel et al., 2014). Nuclear ribosomal cistron sequence data are also frequently used in phylogenetic studies, including fast‐evolving spacer regions (i.e., nrETS and nrITS) and large conserved coding regions (i.e., 18S and 28S) (e.g., Baldwin et al., 1995; Maia et al., 2014; Poczai & Hyvönen, 2010). The least utilized compartment in plant systematics is the mitochondrial genome, presumably due to its relatively slow evolutionary rate, assumed maternal inheritance, assumed shared inheritance with the plastome and occurrence of lateral and intracellular gene transfer (Mower et al., 2012; Rieseberg & Soltis, 1991; Sloan et al., 2009).

Although not without exceptions, the nuclear ribosomal cistron and the organellar genomes are each generally thought to evolve as a single gene due to factors such as concerted evolution of the nuclear ribosomal cistron and uniparental and clonal inheritance of the organellar genomes (Doyle, 1992; Elder & Turner, 1995; Poczai & Hyvönen, 2010; Wolfe & Randle, 2004). These genomic compartments are, therefore, often analyzed in a concatenation framework (e.g., Folk et al., 2016; Govindarajulu et al., 2015; Vargas et al., 2017). However, incongruence within these compartments has been observed; for example, concerted evolution may not be complete, leading to topological incongruence between individual loci of the nuclear ribosomal cistron (Okuyama et al., 2005). Topological incongruence among plastid loci has also been in focus in recent studies, in which it has been suggested that more consideration should be placed on possible incongruence before concatenation of the (widely assumed linked) plastid loci prior to phylogenetic analysis (Gonçalves et al., 2019; Sullivan et al., 2017; Walker et al., 2019; Zhang, Wang, et al., 2020). While stochastic errors due to the paucity of information in individual plastid loci may be responsible for much of the incongruence, other factors (including biological ones such as incomplete lineage sorting [ILS] and recombination) have been considered (Gonçalves et al., 2019; Sullivan et al., 2017; Walker et al., 2019; Zhang, Wang, et al., 2020).

A few recent studies have used genome‐skimming data to reconstruct evolutionary relationships in the coffee family (Rubiaceae), for example, the tribal level sampled Rubiaceae‐wide phylogenies by Rydin et al. (2017) and Wikström et al. (2020), and the Ixoroideae‐focused study by Ly et al. (2020). Those studies showed among other things that datasets from all three genomes could generate overall well‐resolved phylogenetic hypotheses. However, two of the studies also revealed several topological conflicts among results from these genomic datasets (Rydin et al., 2017; Wikström et al., 2020). For example, the mitochondrial tree rejected the monophyly of two of the three subfamilies supported by chloroplast data (Rydin et al., 2017; Wikström et al., 2020).

**FIGURE 1:**
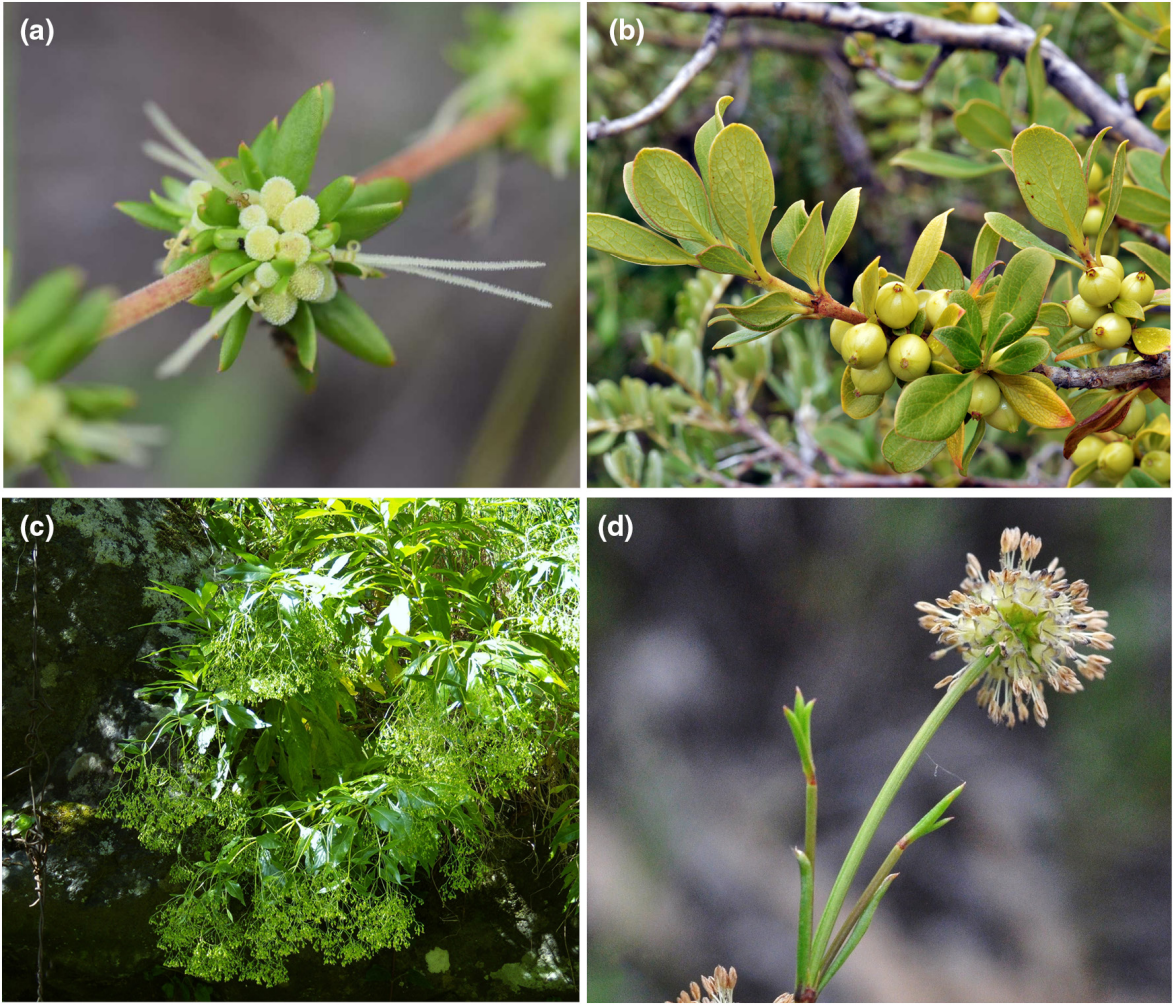
Diversity of Anthospermeae, Rubioideae. (a) *Anthospermum thymoides* subsp. *thymoides*, Madagascar 2017 (photo: E Larsén and O Thureborn), (b) *Coprosma montana*, Hawaii 2013 (photo and ©: JT Johansson, http://angio.bergianska.se), (c) *Phyllis viscosa*, Canary Islands 2009 (photo: R Åkesson, © JT Johansson, http://angio.bergianska.se), (d) *Opercularia vaginata*, Australia 2011 (photo and ©: JT Johansson, http://angio.bergianska.se).

Here we explore the phylogenetic signal of genome‐skimming data for reconstruction of intergeneric relationships within the tribe Anthospermeae (Rubioideae, Rubiaceae) (Figure 1). Its members have some traits that are unusual in the coffee family, that is, wind‐pollinated flowers and a partly temperate distribution. The tribe includes over 200 species in 12 currently recognized genera and can be divided into three main lineages (Thureborn et al., 2019), the South African monogeneric subtribe Carpacocinae (*Carpacoce* Sond.), the African subtribe Anthosperminae (*Anthospermum* L., *Galopina* Thunb., *Nenax* Gaertn., *Phyllis* L.) and a pacific group comprising the subtribe Coprosminae (*Coprosma* J.R.Forst. & G.Forst., *Durringtonia* R.J.F.Hend. & Guymer, *Leptostigma* Arn., *Nertera* Banks ex Gaertn., *Normandia* Hook.f.) and the Australian subtribe Operculariinae (*Opercularia* Gaertn., *Pomax* Sol. x Gaertn.). Anthospermeae are mainly distributed in tropical, subtropical, and temperate regions of the Southern Hemisphere and dominate the Rubiaceae flora in the southwestern Cape Floristic region and New Zealand (Heads, 2017; Puff, 1986; Thureborn et al., 2019). Relaxed molecular clock analyses have provided stem age estimates of c. 43 Ma (Wikström et al., 2015, 2016) and c. 47 Ma (Bremer & Eriksson, 2009), but an ancient Gondwanan origin has also been suggested on the basis of a vicariance model (Heads, 2017).

Two recent molecular phylogenetic studies have generated overall well‐resolved relationships in Anthospermeae; the Anthospermeae‐focused study by Thureborn et al. (2019), which used selected chloroplast and nuclear ribosomal markers, and the Rubioideae‐wide study by Thureborn et al. (2022), in which hundreds of low‐copy nuclear genes were used. However, while taxon sampling was dense in the former study, it was based on a restricted set of molecular markers (two nuclear ribosomal and five chloroplast loci), and some nodes were not statistically supported. The latter study (Thureborn et al., 2022) included one representative for each of 11 of the 12 currently recognized genera and generated a highly supported tree. However, it focused solely on data from the nuclear genome. Thus, evolutionary relationships in this plant group are yet to be studied using phylogenomic data from the mitochondrion, plastome, and nuclear ribosomal cistron. Furthermore, previous work (Thureborn et al., 2019) has indicated several cases of cytonuclear discordance (conflict between organellar and nuclear trees) among (and within) genera in Anthospermeae, although the employed nuclear ribosomal and plastid markers lacked the power to confidently resolve all relationships.

The main aims of the present study are, therefore, to: (1) test whether sequence data obtained via genome skimming (i.e., the two organellar genomes and the nuclear ribosomal cistron) each have the power to recover highly supported phylogenies of Anthospermeae; and (2) investigate the previous indications of cytonuclear discord within the tribe using a substantially larger set of data compared to previous work.

## 2 MATERIALS AND METHODS

### 2.1 Taxon sampling

We sampled herbarium or silica‐dried specimens for one representative of each of the 12 currently recognized genera of Anthospermeae (Thureborn et al., 2019). A set of outgroups were selected based on the results in previous work (Antonelli et al., 2021; Rydin et al., 2017; Thureborn et al., 2019, 2022; Wikström et al., 2020) including representatives for a majority of the other currently recognized tribes of the Spermacoceae alliance. For the nuclear ribosomal dataset, *Faramea* Aubl. (Coussareeae) was also included based on the results in Wikström et al. (2020). Trees were rooted on a representative from the Psychotrieae alliance, *Schizocolea* Bremek. For a list of all taxa used in this study, and voucher and GenBank information, see Table S1. Authors of names follow the International Plant Names Index (IPNI, 2023).

### 2.2 Sequence data generation and preprocessing

Extraction of DNA was performed using the cetyltrimethylammonium bromide method (Doyle & Doyle, 1987). The extracted DNA was purified with a QIAquick polymerase chain reaction (PCR) cleaning kit (Qiagen, Hilden) according to the instructions provided by the manufacturer.

Library preparation and high‐throughput sequencing was carried out at Science for Life Laboratory (SciLifeLab, Solna, Sweden). Library preparation with 350 bp inserts was prepared using the ThruPLEX DNA‐seq library preparation kit from Rubicon (Rubicon Genomics, Ann Arbor, USA) according to the manufacturer's specification. The 12 libraries were multiplexed with 81 other samples and run in three different lanes on the Illumina HiSeq2500 platform (Illumina, San Diego, USA) with 2 × 126 bp paired‐end reads.

The obtained reads were imported into Geneious v11.1.5 (Kearse et al., 2012) and the BBduk v37.64 plugin was used to perform adapter trimming, quality trimming (Q13), and to remove reads shorter than 35 bp. The trimmed reads were used for downstream analyses.

### 2.3 Mitochondrial gene assembly

To mine mitochondrial genes from the read pools, a reference list containing 36 gene regions corresponding to 32 in Rubiaceae conserved protein‐coding genes (including introns) (Rydin et al., 2017) was created, using sequences of *Anthospermum spathulatum* Spreng. (for GenBank accessions, see Appendix S1 in Rydin et al., 2017), *Asclepias syriaca* L. (NCBI reference sequence accession NC_022796) and *Rhazya stricta* Decne. (NCBI reference sequence accession NC_024293) as templates. Each read pool was mapped to the reference list using the Geneious mapper for five iterations and default settings. Mapped reads were subsequently assembled using the Geneious assembler, but sometimes also assembled with the Spades v3.10.0 (Bankevich et al., 2012) and/or Mira v1.1.1 (Chevreux, 2004) plugins in Geneious for verification. Contigs produced by the Geneious and the Mira assembler were visually inspected whereas Spades contigs were quality checked by remapping the full read pool. Occasionally the assemblies resulted in two nonoverlapping contigs. In such cases, the read pools were remapped to those contigs for several rounds (using five iterations each round) until the contigs could be merged and/or the used reads resulted in a complete assembly. Consensus sequences were annotated with annotations from the reference list using the “Annotate from Database” option, and further refined based on alignments with the corresponding reference genes. Introns were also annotated to facilitate extraction with the “Extract annotations” option in Geneious.

### 2.4 Ribosomal cistron assembly

To create a nuclear ribosomal cistron reference (ETS – 18S – ITS1 – 5.8S – ITS2 – 26S) we combined the 18S and 26S genes *of Asclepias syriaca* (NCBI GenBank accession JF312046) with ITS (ITS1 – 5.8S – ITS2) (NCBI GenBank accession MK141304) and ETS (NCBI GenBank accession MK141214) sequences of *Durringtonia paludosa* R.J.F.Hend. & Guymer. This sequence was used to create a nuclear ribosomal subset of reads by mapping each sample to the reference with the Geneious read mapper, using five iterations and default settings. The resulting read pools where de novo assembled using Spades within Geneious. The resulting contigs where then merged with the Geneious assembler, creating nuclear ribosomal draft sequence for each sample. The full read pools where then remapped to the draft sequence using five iterations. The consensus sequences were annotated based on the alignment with outgroup sequences.

### 2.5 Plastome assembly

To isolate chloroplast reads, the full read pools were mapped to a reference database that contained the 11 chloroplast genomes of species of the Spermacoceae alliance produced by Wikström et al. (2020). Mapping was performed using the Geneious mapper with default settings and five iterations. Then the mapped reads were de novo assembled using Spades within Geneious. De novo contigs were subject to iterative mapping to further validate and quality‐control the contigs. This allowed for coverage‐based verification and the construction of longer contigs, which were (where possible) merged using the Geneious assembler. The contigs were subsequently ordered in their most likely orientation using the Geneious plugin Mauve v2.3.1 (Darling et al., 2004) with the Mauve Contig Mover algorithm (Rissman et al., 2009) with *Argostemma hookeri* King (NCBI GenBank accession KY378693) set as reference genome and a consensus sequence with a spacer of 100 Ns between each contig was saved. The full read pools were subsequently mapped to the draft genomes to identify and correct errors and calculate coverage. Gaps were filled with “?” characters. The consensus sequences were initially annotated with annotations from the references using the “Annotate from Database” option in Geneious and further refined based on alignments with other closely related chloroplast genomes. Introns and intergeneric spacers were also annotated to facilitate extraction with the “Extract annotations” option in Geneious. The boundaries of the inverted repeat (IR) units were identified by using the “Find Repeats” option in Geneious. To create a circular draft plastid genome sequence, repeats found at the end of the consensus sequence were deleted, and the IR region was copied, reverse‐complemented, and concatenated with the consensus.

To identify putative plastome rearrangements the newly generated plastomes were aligned with the plastome of *Coffea arabica* L. (NCBI GenBank accession MK875244) as reference using Mauve with the progressiveMauve algorithm (Darling et al., 2010) within Geneious. *Coffea arabica* was used as a reference as it has the same organization as the inferred ancestral angiosperm plastome (Samson et al., 2007). One IR from each plastome was removed before alignment.

### 2.6 Alignment, identification of putative small hairpin inversions and cleanup

All loci were separately aligned with MAFFT v7.470, using the G‐INS‐i with a variable scoring matrix algorithm to control for overalignment (Katoh & Standley, 2016) within the alignment editor Aliview v1.26 (Larsson, 2014). For protein‐coding genes (PCGs), nucleotides were first translated to amino acids and then back‐translated to nucleotides. PCGs that contained putative frameshifts and/or premature stop codons were first put in frame using MACSE v2.03 (Ranwez et al., 2011, 2018). Stop codons were removed. Strings of 100 Ns annotated as gaps of unknown length in GenBank were removed prior to alignment. The MACSE algorithm was executed within the software PhyloSuite v1.2.2 (Zhang, Gao, et al., 2020).

Short inversions associated with hairpins are common features in non‐coding DNA regions and can be very liable to both parallelism and reversal within even closely related groups and may severely mislead phylogenetic inference (Kelchner, 2000; Kelchner & Wendel, 1996; Kim & Lee, 2005; Quandt et al., 2003). The alignments were visually inspected for short inversions associated with hairpins (three or more neighboring nucleotide substitutions flanked by inverted repeats) in non‐coding DNA as well as coding regions. For validation of putative hairpin inversions, the region containing the inversion region and its flanking bases were folded with the program RNAfold from the Vienna RNA package (Hofacker, 2003) implemented in Geneious to check for the presence of a stem‐loop structure. In some species, the putative hairpin structure had been lost or disrupted. In total, 17 (four in the mitochondrial dataset and 13 in the plastid dataset; Table S2) putative inversion regions were either deleted or alternatively reverse complemented to not disrupt the open reading frame of PCGs.

To limit the number of uninformative loci we removed two very short (below 11 bp) plastid intergenic spacer regions (IGSs). For the same reason, we concatenated all rRNA alignments into a single marker, and all tRNA alignments into a single marker. The *trnS* ^*GGA*^ – *rps4* IGS was removed from downstream phylogenetic analyses because we detected putative insertions of mitochondrial DNA. Excluding the *trnS* ^*GGA*^ – *rps4* IGS alignment, we used 202 plastid markers (77 PCGs, 21 introns, 102 IGSs, one tRNA and one rRNA), 47 mitochondrial markers (31 PCGs and 16 introns) and six nrDNA markers (three spacer regions and three rRNAs). However, for comparison, we also conducted an analysis where the plastid *trnS* ^*GGA*^ – *rps4* IGS alignment was included.

### 2.7 Congruence test

Intragenomic congruence among loci within the mitochondrial dataset, the chloroplast dataset, and the nuclear ribosomal cistron dataset, respectively, was examined using the hierarchical likelihood ratio test implemented in Concaterpillar v1.8a (Leigh et al., 2008), which internally used RAxML v7.3 with the GTRGAMMA substitution model for maximum‐likelihood phylogenetic inference. Sequence alignments for sets of regions of each genomic compartment were concatenated according to different strategies. For each genomic compartment, we used the concatenation of the largest congruent set(s) identified by Concaterpillar. In case not all regions within each compartment resulted in one congruent set we also combined all regions for that compartment (combined dataset).

### 2.8 Phylogenetic analyses

We used IQ‐TREE v1.6.8 (Nguyen et al., 2015) implemented in PhyloSuite for phylogenetic inference in the maximum likelihood (ML) framework. The datasets were analyzed both as unpartitioned and partitioned. For the unpartitioned analyses we used the GTR + G model and for the partitioned analyses we used ModelFinder (Kalyaanamoorthy et al., 2017) implemented in IQ‐TREE to find an optimally partitioned model according to the Bayesian information criterion (Schwarz, 1978) for each dataset. For the plastid data, each intron, IGS, and PCG, as well as the grouped tRNAs and rRNAs were given as input for the model selection before the partitioning search. For the nuclear ribosomal data, each spacer and each coding region were given as input. For the mitochondrial data, each intron and each PCG was given as input. We also used RY‐coding, an approach that divides the four nucleobases into two character states (purines and pyrimidines), to limit the effect of potential biases caused by saturation and base composition in sequence data (Ishikawa et al., 2012; Phillips & Penny, 2003; Praz & Packer, 2014). The RY‐coded matrices were analyzed as unpartitioned. Branch support was assessed using the three approaches implemented in IQ‐TREE: ultrafast bootstrap approximation (UFBoot) with 2000 replicates (Hoang et al., 2018; Minh et al., 2013), SH‐like approximate likelihood ratio test (SH‐aLRT) with 2000 replicates (Guindon et al., 2010) and the approximate Bayes test (aBayes) (Anisimova et al., 2011).

## 3 RESULTS

### 3.1 Mitochondrial gene assembly

Mitochondrial assembly statistics are found in Table S3. Of the 36 targeted mitochondrial gene regions, 35 were assembled for all taxa; *rps7* was not possible to assemble in any of the investigated taxa. Average coverage ranged between 19.3× (*Coprosma*) and 244.6× (*Normandia*). Assembled total gene length ranged between 50,913 bp (*Anthospermum*) and 61,205 bp (*Opercularia*). Based on coverage there was no indication that the assembled gene regions would represent nuclear sequences of mitochondrial origin (NUMTs). All assembled regions were used for phylogenetic analyses.

### 3.2 Nuclear ribosomal cistron assembly

Nuclear ribosomal cistron assembly statistics are found in Table S3. Assemblies of large portions of the nuclear ribosomal cistron were successful for all investigated taxa with average coverages ranging between 473.3× (*Galopina*) and 1865.1× (*Anthospermum*). The recovered length of the nuclear ribosomal cistron ranged between 6320 bp (*Anthospermum*) and 8770 bp (*Nertera*). These differences in assembled lengths were mainly due to variation in recovery of the intergenic spacer region (IGS), which consists of the external transcribed spacer (ETS) and the non‐transcribed spacer (NTS) regions. For phylogenetics we used a conserved region of the ETS (ca 530 bp), and the complete 18S, 26S, 5.8S, ITS1, and ITS2 regions.

### 3.3 Plastome assembly

Plastome assembly statistics are found in Table S3. Complete or near complete plastid genome assemblies were achieved for all taxa with average coverage ranging between 126.5× (*Coprosma*) and 1444.5× (*Phyllis*). The assembled and annotated sequences had the typical quadripartite organization of angiosperms, with a large single copy region (LSC), a small single copy region (SSC) and the inverted repeat region (IR). The assembled plastome length ranged between 153,709 bp (*Phyllis*) and 156,932 bp (*Nertera*). For phylogenetic analyses we extracted 77 PCGs, 30 tRNAs, 4 rRNAs along with the majority of non‐coding regions, comprising 21 introns, and 103 IGSs (102 IGSs + the *trnS* ^*GGA*^ – *rps4* IGS including its putative mitochondrial insertion). The putative pseudogenes *infA* and *rpl33* were included as part of the *rpl36* – *rps8* and *psaJ* – *rps18* IGS regions, respectively.

**FIGURE 2:**
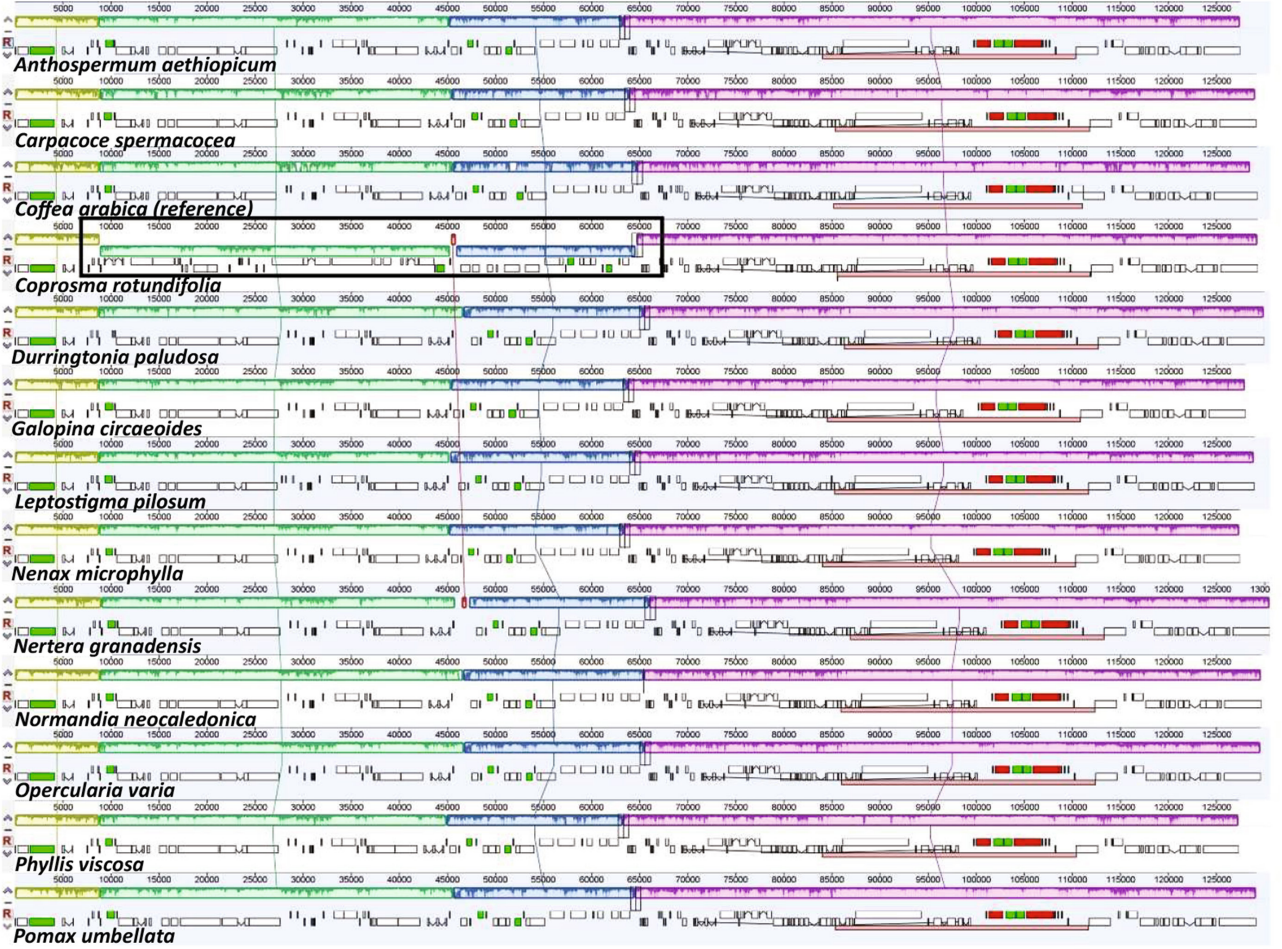
Alignment of the twelve Anthospermeae plastomes sequenced in this study, and the reference plastome *Coffea arabica* (produced using Mauve Genome Alignment v2.3.1). Corresponding colored boxes connected by lines denote locally collinear blocks (LCBs), which represent presumably homologous regions. Sunken blocks indicate an inverted orientation relative to the reference. The putative inversions in *Coprosma rotundifolia* are highlighted with a black rectangle. Heights of vertical bars within LCBs indicate relative sequence similarity level at that position.

Relative to the *Coffea arabica* plastome the newly sequenced plastomes were unrearranged, with the exception of two large inversions in *Coprosma rotundifolia* A.Cunn. (Figure 2). The first inversion (approximately 36‐kb) spans *trnS* ^*GGA*^ and *trnS* ^*GCU*^, and the second inversion (approximately 19‐kb) spans *petA* and *rps4*. The two inversions result in that *psbI* neighbors *trnS* ^*GGA*^, *trnS* ^*GCU*^ neighbors *petA*, and *rps4* neighbors *psbJ*. Both inversions were found within a gapless sequence of the near‐complete *Coprosma rotundifolia* plastome assembly.

### 3.4 Dataset characteristics and congruence test

The concatenated plastid alignment consisted of 202 markers and a total of 149,391 bp, with 10,820 variable sites, and 3262 parsimony informative sites (the number of variable and parsimony informative sites reported here were calculated with the outgroups excluded). The inclusion of the *trnS* ^*GGA*^ – *rps4* IGS added 4629 bp, of which 511 were variable, and 134 were parsimony informative. The concatenated mitochondrial alignment consisted of 70,519 bp, with 1417 variable sites, and 307 parsimony informative sites. The final concatenated nuclear ribosomal alignment consisted of 6545 bp, with 672 variable sites, and 330 parsimony informative sites.

Based on the hierarchical ratio tests conducted using Concaterpillar, the null hypothesis of congruence among loci was not rejected for the mitochondrial loci, nor for the nuclear ribosomal loci. Analyses of the mitochondrial and nuclear ribosomal datasets were thus only conducted on the complete datasets.

In contrast, the null hypothesis of congruence among all loci was rejected for the plastid dataset, resulting in nine statistically distinct subsets of congruent loci (Table S4). The nine subsets included 89, 79, 25, 2, 3, 1, 1, 1, and 1 loci, respectively, and contained 76,684, 51,945, 19,296, 210, 287, 117, 111, 101, and 640 bp, respectively. Subset 1 (comprising 89 loci) consisted of 76,684 bp, with 5426 variable sites, and 1609 parsimony informative sites. Subset 2 (comprising 79 loci) consisted of 51,945 bp with 4283 variable sites, and 1329 parsimony informative sites. Subset 3 (comprising 25 loci) consisted of 19,296 bp with 1071 variable sites, and 314 parsimony informative sites. Subset 4 (two loci) consisted of 210 bp with 5 variable sites, and 1 parsimony informative site. Subset 5 (three loci) consisted of 287 bp with 9 variable sites and 3 parsimony informative sites. Subset 6 (one locus) consisted of 117 bp with 2 variable sites and 1 parsimony informative site. Subset 7 (one locus) consisted of 111 bp with 5 variable sites and 2 parsimony informative sites. Subset 8 (one locus) consisted of 101 bp with 8 variable sites and 2 parsimony informative sites. Subset 9 (one locus) consisted of 640 bp with 11 variable sites and 1 parsimony informative site. Information regarding loci included in each dataset is found in Table S4. Because of the few variable and parsimony informative sites in most of these subsets (subsets 4–9), only the three largest plastid subsets (subsets 1–3) were used for phylogenetic analyses.

### 3.5 Mitochondrial intron length variation

Large length variation (>1000 bp) was detected in introns of four mitochondrial genes, namely *ccmFc* (922–2628 bp), *nad2* intron 3 (1558–8718 bp), *nad4* intron 3 (1846–3151 bp), and *nad5* intron 4 (1088–3917 bp). Autapomorphic insertions in the *ccmFc* intron (*Phyllis*), *nad4* intron 3 (*Opercularia*) and *nad5* intron 4 (*Opercularia*) explained most of the length variation in those regions; however, some of these insertions show strong similarity to nuclear or plastid DNA regions. The *ccmFC* intron was greatly expanded in *Phyllis viscosa* Christ, and a MegaBLAST search of the inserted region revealed matches to a nuclear‐encoded shaggy‐related protein kinase alpha sequence (ca 1500 bp) of *Coffea* species and other angiosperms. The *nad4* intron‐3 was greatly expanded in *Opercularia*, and a MegaBLAST search of the inserted region comprised mitochondrial hits. The *nad5* intron‐4 was greatly expanded in *Opercularia*, and MegaBLAST searches showed both mitochondrial and plastid hits. The plastid hits included strong matches to a region covering the 3′ end of *ycf1* (ca 330 bp) and the *ycf1* – *rps15* IGS (ca 100 bp). The *nad2* intron‐3 was much longer in the Pacific subclade (2917–8718 bp) compared to the remaining Anthospermeae (1879–1931 bp) and outgroups (1558–1978 bp). MegaBLAST searches of the inserts almost exclusively resulted in matches to mitochondrial regions of various angiosperms.

### 3.6 Sequences of mitochondrial origin in the plastome

The plastid *trnS* ^GGA^ – *rps4* intergenic spacer was highly variable in length, ranging from 267 to 2515 bp. The length ranged between 1206 and 2515 bp long in the members of the Pacific subclade (species of *Coprosma* lack the *trnS* ^*GGA*^ – *rps4* IGS); however, it varied between 267 and 329 bp in length in the remaining Anthospermeae and in outgroups. For the sequences retrieved from members of the Pacific subclade, between 163 and 347 of the last 3′ bases (i.e., upstream of *rps4*) of the long *trnS* ^GGA^ – *rps4* spacer of each species showed high similarities with the *trnS* ^GGA^ – *rps4* IGS of remaining Anthospermeae and outgroups based on MSAs and MegaBLAST searches. MegaBLAST searches against the NCBI nr database of the remaining sequence of *Leptostigma* (positions 1–964), *Nertera* (1–2053), *Normandia* (1–1770), *Durringtonia* (1–1698), *Pomax* (1–955) and *Opercularia* (1–1657) revealed multiple hits (between 101 and 493 bp in length) to mitochondrial genomes of various angiosperms, with a total query coverage of 43.7% (897 bp) for *Nertera*, 12.7% (122 bp) for *Leptostigma*, 22.4% (214 bp) for *Pomax*, 47.8% (846 bp) for *Normandia*, 51.6% (876 bp) for *Durringtonia*, and 52.0% (862 bp) for *Opercularia*. Only hits longer than 100 bp were considered.

Due to plastome rearrangements in *Coprosma*, sequences presumably homologous to the *trnS* ^GGA^ – *rps4* IGS are instead found in the *trnS* ^GCU^ – *petA* and *rps4* – *psbJ* IGSs, which are unique to *Coprosma*. MegaBLAST searches and MSAs of *trnS* ^GCU^ – *petA* (1078 bp) given minor uncertainties in delimiting the boundaries revealed that 427 of the 3′ bases show high similarity to the 5′ end of the *petA* – *psbJ* IGS of remaining Anthospermeae and outgroups. The remaining sequence did not return any hits but a 555 bp 5′ portion aligns with non‐plastid regions of the *trnS* ^GGA^ – *rps4* of the others. MegaBLAST searches and MSAs of *rps4* – *psbJ* showed (given minor uncertainties in delimiting the boundaries) that a 5′ portion (758 bp) shows high similarity with the plastid region (292 bp) of remaining Anthospermeae and outgroups, and the adjacent non‐plastid region of *Nertera* (466 bp) of the 5′ end of *trnS* ^GGA^ – *rps4* and 486 bp of the 3′ end align with the 3′ end of *petA* – *psbJ* of the remaining Anthospermeae and outgroups.

### 3.7 Phylogenomic results and congruence

#### 3.7.1 Mitochondrial data

There were no topological conflicts between the tree topologies of Anthospermeae resulting from the partitioned, unpartitioned, and RY‐coded analyses of the mitochondrial dataset (Figure S1).

#### 3.7.2 Nuclear ribosomal data

When comparing tree topologies from the partitioned, unpartitioned, and RY‐coded analysis of nuclear ribosomal data there were some unsupported differences between the RY‐coded and non‐RY‐coded derived trees (Figure S2).

#### 3.7.3 Plastid data

There were no conflicts between tree topologies resulting from the partitioned, unpartitioned, and RY‐coded analysis of plastid data (Figure S3) and subsets of plastid data (Figures S4–S6) except for plastid subset 1 (Figure S4), where one unsupported difference between RY‐coded and non‐RY‐coded trees was revealed (regarding the positions of *Durringtonia* and *Opercularia*).

#### 3.7.4 Topological results supported in analyses of all three genomic compartments

Unless otherwise stated we here refer to the results obtained with the best‐fit partitioned model. For support thresholds we use at least 95 for the ultrafast bootstrap and at least 80 for SH‐aLRT and at least 95 for aBayes based on recommendations in the literature (Anisimova et al., 2011; Guindon et al., 2010; Minh et al., 2013). We chose to consider a node well supported if at least two of these three thresholds were reached.

**FIGURE 3:**
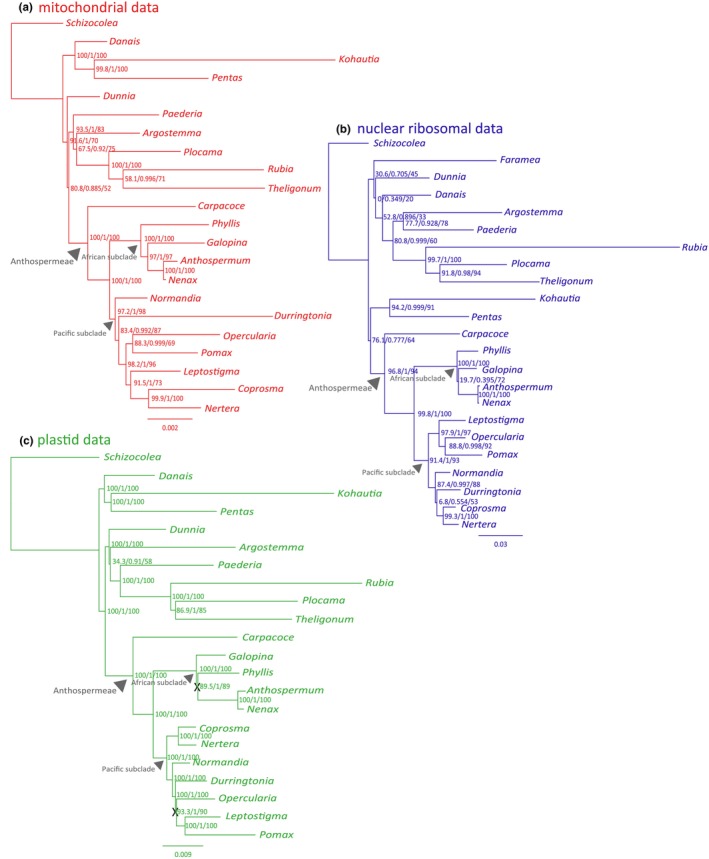
Phylogenetic results based on maximum likelihood analyses (partitioned datasets). (a) mitochondrial data, (b) nuclear ribosomal data, and (c) plastid data. Support values at nodes represent ultrafast bootstrap (UFboot)/approximate Bayes (aBayes)/approximate likelihood ratio test (SH‐aLRT). The clades Anthospermeae, the African subclade, and the Pacific subclade are discussed in the text. The X:s denote conflicting intra‐plastome signal discussed in the text.

**FIGURE 4:**
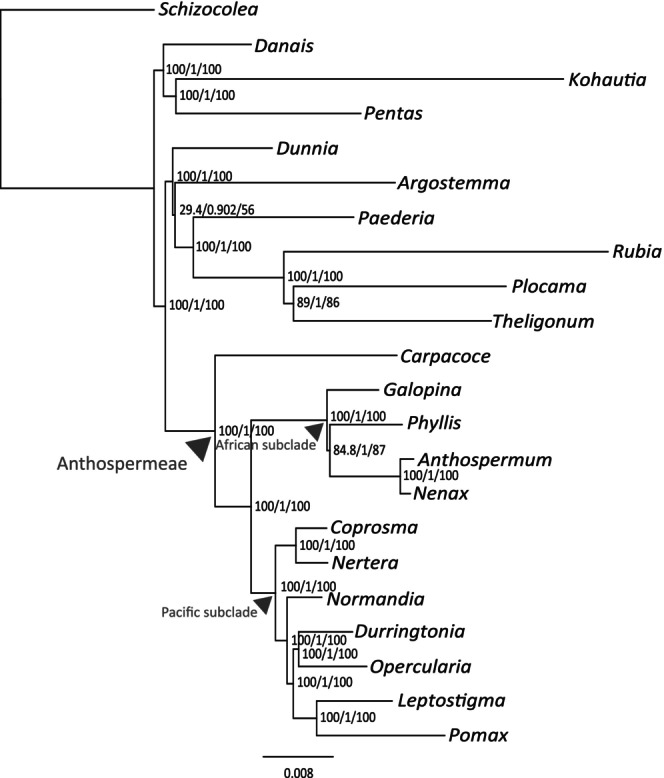
Phylogenetic results based on the combined unpartitioned plastid dataset including the *trnS* ^*GGA*^ – *rps4* region, in which the genera of the Pacific clade have an insertion of putative mitochondrial origin (except *Coprosma*, see the text for more information).

Anthospermeae was strongly supported as monophyletic in all but one of our analyses. The exception was the low support for Anthospermeae in the analysis of RY‐coded nuclear ribosomal data (Figure S2b). The South African genus *Carpacoce* is sister to the remaining members of Anthospermeae, which in turn were divided into two major sister clades hereafter referred to as the African subclade (subtribe Anthosperminae) and the Pacific subclade (subtribes Coprosminae and Operculariinae). These clades were well supported in all analyses (Figures 3 and 4; Figure S1–S6). The respective sister relationships *Anthospermum* – *Nenax* and *Coprosma* – *Nertera* were strongly supported in all analyses except for the *Coprosma* – *Nertera* sister relationship in the analysis of the RY‐coded plastid subset 3 (Figure S6b).

#### 3.7.5 Topological incongruences

##### The African subclade of Anthospermeae

Results based on mitochondrial data and nuclear ribosomal data are congruent regarding topological results in the African subclade, with *Phyllis* and *Galopina* being successive sisters to the *Anthospermum* – *Nenax* clade (Figure 3a,b). In contrast, plastid data resolve *Galopina* as sister to the remaining African subclade, followed by *Phyllis* sisters to the *Anthospermum* – *Nenax* clade (Figure 3c). However, the support for the (*Galopina* (*Anthospermum* – *Nenax*)) clade is low in the analysis of nuclear ribosomal data (Figure 3b), and the RY‐coded analysis of nuclear data (Figure S2b) resolved *Phyllis* and *Galopina* as sisters (no support). It should further be noted that analyses of plastid subset 2 (Figure S5) resolve *Galopina* and *Phyllis* as sisters, mostly with high support.

##### The Pacific subclade of Anthospermeae

The topology within the Pacific subclade differs considerably between results from the three genomic compartments (Figure 3). Mitochondrial data resolve *Normandia* as sister to the remaining clade, followed by *Durringtonia* sister to a clade comprising *Opercularia* – *Pomax* and *Leptostigma* sister to *Coprosma* – *Nertera* (Figure 3a). Nuclear ribosomal data resolve a (*Leptostigma* (*Opercularia* – *Pomax*)) clade sister to a clade comprising *Normandia* sister to a (*Durringtonia* (*Coprosma* – *Nertera*)) clade (Figure 3b). Based on plastid data, the *Coprosma* – *Nertera* clade is sister to the remaining Pacific subclade, within which *Normandia* is sister to remaining genera, followed by *Durringtonia* sister to a clade comprising *Opercularia* sister to *Leptostigma* and *Pomax* (Figure 3c). All these results are mostly well supported. Based on mitochondrial data, these results are consistent in all analyses (of partitioned, unpartitioned, and RY‐coded data, Figure S1). Based on nuclear ribosomal data, there is an unsupported difference in the *Normandia* – *Durringtonia* – *Coprosma* – *Nertera* clade between results based on RY‐coded and non‐RY‐coded data (Figure S2). For plastid data, analyses of subsets 2 and 3 (Figures S5, S6) consistently result in the same topology as do analyses of the entire plastome dataset (Figure 3c; Figure S3), that is, *Normandia*, *Durringtonia* and *Opercularia* being successive sisters to a *Leptostigma* – *Pomax* clade. In contrast, plastid subset 1 displays conflicting topologies between results from RY‐coded and non‐RY‐coded data (Figure S4). While the results based on RY‐coded data of plastid subset 1 are the same as stated above (Figure S4b), the non‐RY‐coded data of the same subset instead returns a sister relationship between *Durringtonia* and *Opercularia* (Figures S4a,c). These relationships are not well supported in the analyses of subset 1. However, the *Durringtonia* – *Opercularia* clade is strongly supported when analyzing the entire plastome dataset, including the *trnS* ^GGA^ – *rps4* region (Figure 4), which was otherwise removed from all analyses due to its long insertion of putative mitochondrial origin.

## 4 DISCUSSION

### 4.1 Topological incongruence in Anthospermeae

The backbone phylogeny of the tribe Anthospermeae is resolved and strongly supported in results based on data from each of the three genomes (Figure 3). The tribe is monophyletic and *Carpacoce* is sister to the remaining clade, which comprises an African subclade and a Pacific subclade. Results within the African and Pacific subclades as retrieved based on data from the three genomic compartments are, however, not in full agreement (Figure 3). Within Anthospermeae, this is most notable in the Pacific subclade (Figure 3). There are also examples of intragenomic conflict within the plastome (Figures 3c, 4; Figures S4–S6). Statistically well supported topological incongruence can have several explanations, for example, technical reasons such as inadequate taxon sampling, modeling error and sampling error (Rieseberg & Soltis, 1991; Wendel & Doyle, 1998). In order to explore possible reasons for detected topological incongruence, we investigated and analyzed our datasets in several ways.

We were not able to find any obvious signs of severe modeling or sampling error. We challenged the phylogenetic results from each genomic compartment by using alternative data coding (RY‐coding) that may decrease the biasing effect of nucleotide saturation and composition (although at the expense of loss of phylogenetic signal) (Phillips & Penny, 2003; Ruhfel et al., 2014). Our topological results are robust to these RY‐coding experiments in the sense that although both increases and decreases in statistical support were observed, the resulting topologies retrieved from the RY‐coded dataset are mostly consistent with those inferred from the non‐RY‐coded datasets (Figures S1–S6).

We further challenged our results by conducting hierarchical ratio tests. For nuclear ribosomal cistron data and mitochondrial data, the null hypothesis of congruence among all loci could not be rejected and only the respective complete datasets were analyzed for those compartments. No previous study has analyzed the phylogeny of Anthospermeae using mitochondrial data, but regarding results based on nuclear data there is a single topological conflict between results of the present study and those in Thureborn et al. (2022), in which the monospecific Australian genus *Durringtonia* was sister to the remaining members of the Pacific clade, whereas analyses of nuclear data in the present study place *Durringtonia* in a clade with *Normandia* and the *Coprosma* – *Nertera* clade. A difference between the two studies (except regarding the taxon sampling) is that Thureborn et al. (2022) utilized data from hundreds of low‐copy nuclear loci, whereas the present study uses data from the nuclear ribosomal cistron. The results in Thureborn et al. (2022) indicated relatively high levels of ILS‐induced gene tree heterogeneity in Rubioideae (including Anthospermeae), and ILS may also explain the incongruence between nuclear ribosomal DNA used in the present study and the species tree estimate in Thureborn et al. (2022).

For plastid data, the null hypothesis of congruence among all loci was rejected, as assessed using hierarchical likelihood ratio tests, resulting in nine subsets of congruent loci (Table S4). We considered three of those sets (those that contained more than 1–3 loci) and conducted additional analyses of those three subsets of plastid loci (Figures S4–S6). Our analyses of those statistically distinct subsets showed some cases of intragenomic topological incongruence, for example, between the plastid subsets 1 and 2 (Figures S4, S5). If plastid genes were fully linked, this finding could indicate modeling error. However, the overall robustness to different partitioning schemes and the RY‐coding experimentation may indicate biological reasons for the observed incongruences. We also find (unsupported) conflict between analyses of RY‐coded and non‐RY‐coded data, for example, for subset 1. We find no obvious (e.g., functional) pattern that could explain the grouping of loci into these subsets; there are, for example spacers, introns and protein‐coding genes in all three subsets (Table S4), although it could be mentioned that many genes of the small single copy region of the plastome are found in plastid subset 1.

Comparing our results from plastid data (Figure 3c) with those of the previous study of the phylogeny of Anthospermeae by Thureborn et al. (2019), we conclude that results are congruent with one exception: the sister relationship between *Galopina* and *Phyllis* found in Thureborn et al. (2019) differs from the successive sister relationships of *Galopina* and *Phyllis* to the *Anthospermum* – *Nenax* clade retrieved in the present study. The support for the *Galopina* – *Phyllis* clade was not strong in Thureborn et al. (2019), but the same topology is highly supported in one of our analyses of plastid data, that of subset 2 (Figure S5). Of the plastid markers used in Thureborn et al. (2019), three (*rbcL* PCG, *trnT* – *trnL* IGS, *atpB* – *rbcL* IGS) are here included in subset 2, whereas two (*trnL* – *trnF* IGS, *rps16* intron) are included in subset 1 and one (*ndhF* PCG) in subset 3 (Table S4). This may explain the result patterns in both studies including the low support for the *Galopina* – *Phyllis* clade in Thureborn et al. (2019), and indicates further support for the presence of intragenomic conflict in the plastome in our study group.

### 4.2 Biological reasons for intergenomic topological incongruence

Mitochondrial data have been much less utilized in plant phylogenetic studies compared to nuclear ribosomal and plastid data, and one reason is the assumed shared maternal inheritance of the organellar genomes in most plant groups (Greiner et al., 2015; McCauley, 2013; Mower et al., 2012). In agreement with previous work (Folk et al., 2016; Govindarajulu et al., 2015; Rydin et al., 2017), our study shows, however, that phylogenies derived from mitochondrial and plastid data are not always congruent. In case of hybridization events, such patterns are expected if the organelles exhibit different parental modes of inheritance, as, for example, in Pinaceae where plastids are paternally inherited and mitochondria are maternally inherited (Wang & Wang, 2014). In angiosperms, low levels of paternal transmission (leakage) of organelles may be common, especially in gynodioecious plants (which occur in Anthospermeae) (Azhagiri & Maliga, 2007; Mandel et al., 2012; McCauley, 2013; Wade & McCauley, 2005). Coupled with interspecific hybridization, leakage may result in discordant organellar histories. For example, different modes of inheritance was invoked as an explanation for the differing organellar phylogenies in *Fragaria* L. (Rosaceae) (Govindarajulu et al., 2015).

Interspecific hybridization followed by introgression may also result in conflicts between organellar and nuclear trees when the organellar genome(s) of one species are replaced by that of another and the nuclear genome is kept more or less intact (Stegemann et al., 2012; Tsitrone et al., 2003), a phenomenon also known as organelle capture. Organelle capture (plastid or mitochondrial) is often suggested as explanation for cytonuclear discordance (Folk et al., 2016; Nge et al., 2021; Yang et al., 2021) and may explain the conflicts observed in the present study as well. However, other processes, such as incomplete lineage sorting (Maddison, 1997) cannot be ruled out at this point as they may result in similar patterns as hybridization.

### 4.3 Intragenomic incongruence in the plastid data

The chloroplast genome has long been considered to be essentially non‐recombinant and uniparentally inherited (Birky, 1995; Vogl et al., 2003). Plastome loci are, therefore, assumed to share the same phylogeny (which is a key assumption when using concatenation approaches), also in case of hybridization. Plastomes are, therefore, often treated as a single locus in phylogenetic analysis (Doyle, 1992; Gitzendanner et al., 2018). In that respect incongruence among plastid loci is surprising, but has nevertheless been previously shown in several different angiosperm groups (Erixon & Oxelman, 2008; Gonçalves et al., 2019; Sullivan et al., 2017; Walker et al., 2019; Zhang, Sun, et al., 2020; Zhang, Wang, et al., 2020), and there are exceptions to the assumptions of chloroplast inheritance and recombination (Wolfe & Randle, 2004). The potential for biparental transmission of plastid DNA has been indicated in several angiosperm families (Corriveau & Coleman, 1988; Zhang, et al., 2003). Heteroplasmy as a result of biparental inheritance has also been reported (Ramsey & Mandel, 2018) and provides the potential for inter‐plastome recombination. Interspecific hybridization and recombination was suggested to be the processes responsible for the chimeric plastomes and incongruent phylogenies detected in Sileneae (Caryophyllaceae), *Picea* (Pinaceae) and the *Cupressus* – *Juniperus* – *Xanthocyparis* complex (Cupressaceae) (Erixon & Oxelman, 2008; Sullivan et al., 2017; Zhu et al., 2018). Although plastomes have been considered to be practically immune to foreign DNA there are documented cases of DNA transfer from the mitogenome to the plastome (Gandini & Sanchez‐Puerta, 2017; Smith, 2014). Horizontal gene transfer followed by gene conversion between native and foreign copies have created chimeric mitochondrial genes in angiosperms, which may cause phylogenetic artifacts (Hao et al., 2010; Hao & Palmer, 2009, 2011), and it is plausible that such processes may have been acting on the plastome as well (Straub et al., 2013).

### 4.4 Mitochondrial insertion in the plastomes of the Pacific subclade

We detected probable mitochondrial inserts in the plastome in all the genera of the Pacific subclade of Anthospermeae. The inclusion/exclusion of this region in datasets had a decisive impact on topological results (compare the positions of *Durringtonia* and *Opercularia* in Figures 3c, 4). DNA transfer from the mitogenome to the plastome is considered a rare event (Gandini & Sanchez‐Puerta, 2017; Smith, 2014); such transfers have previously only been reported in a few flowering plant families and always in IGS regions (Burke et al., 2016, 2018; Goremykin et al., 2009; Iorizzo, Grzebelus, et al., 2012; Iorizzo, Senalik, et al., 2012; Rabah et al., 2017; Raman et al., 2019; Straub et al., 2013; Wysocki et al., 2015). The plastome, in contrast to the mitogenome, appears to lack an active DNA uptake mechanism and is thus believed to be almost impermeable to the incorporation of foreign DNA (Iorizzo, Grzebelus, et al., 2012; Richardson & Palmer, 2006; Smith, 2011). However environmental stresses could facilitate transfer of foreign DNA into the plastid (Cerutti & Jagendorf, 1995), and once inside the plastid it could be inserted into the plastome via homologous recombination (Maréchal & Brisson, 2010; Rabah et al., 2017). Mitochondrial insertions have also been observed in plastomes of Orobanchaceae and Lamiaceae based on BLAST analyses of publicly available plastomes and mitogenomes, but miss‐assembly could not be ruled out as the original reads were not available (Gandini & Sanchez‐Puerta, 2017). Here, the average sequencing depth of those regions were uniform and similar compared to the rest of the plastome, and much higher than the average coverage of the mitochondrial genes (Table S3, Figure S7). There is thus no reason to believe that the insertions detected here are the result of miss‐assemblies. Interpreting the distribution of the inserts and the rarity of mitogenome‐to‐plastome transfer, the most parsimonious scenario for the mitochondrial insertion would be that it occurred as a single event in the common ancestor of the Pacific subclade.

In contrast to the presumably rarely occurring transfer of DNA from the mitogenome to the plastome, DNA transfer from the plastome to the other genomic compartments, and between the nuclear genome and the mitogenome are rather frequent in angiosperms and other seed plants (Bock, 2010; Keeling & Palmer, 2008; Renner & Bellot, 2012; Richardson & Palmer, 2006; Sloan & Wu, 2014; Smith, 2011; Zhao et al., 2018). Nuclear genome‐to‐mitogenome transfer is mostly known to concern transposable elements and to a lesser extent exons, which are often inferred to be pseudogenes (Qiu et al., 2014). However, the insertion in the *ccmFc* intron in *Phyllis viscosa* detected here interestingly includes a sequence with an intact coding frame.

## 5 CONCLUSIONS

Our work shows that data from each of the three genomic compartments have the power to resolve the backbone phylogeny of Anthospermeae. Most results retrieve strong statistic support and are consistent among results from the three genomes. *Carpacoce* is sister to the remaining Anthospermeae, which comprise two groups: the African subclade (*Galopina*, *Phyllis*, *Anthospermum*, *Nenax*) and the Pacific subclade (*Coprosma*, *Nertera*, *Normandia*, *Durringtonia*, *Opercularia*, *Leptostigma*, *Pomax*) of Anthospermeae. However, some results within these respective subclades differ among results from the three genomes (Figure 3a–c), and while some of the differing nodes are poorly supported, others are strongly supported.

Furthermore, the congruence tests rejected the null hypothesis of congruence among all plastid loci, resulting in a division of the plastome into several subsets of congruent loci. Analyzing these statistically distinct subsets separately revealed topological incongruence within the African and the Pacific subclades of Anthospermeae, for example, between results from plastid subsets 1 and 2 (Figures S4, S5). The root of this phylogenomic discord is not clear. While model misspecification and sampling error is possible, hybridization, introgression, horizontal gene transfer and chloroplast recombination, as well as incomplete lineage sorting, are among other plausible explanations for the observed topological incongruences.

Finally, we detected a (rarely reported) mitochondrial insertion event in the plastid IGS *trnS* ^GGA^ – *rps4* region, which may be a synapomorphic feature for the Pacific subclade of Anthospermeae. Interestingly, the inclusion/exclusion of this gene region from phylogenetic analyses strongly influences the topological result in the Pacific clade. When excluded, most analyses of plastid data resulted in a grade of taxa (*Normandia*, *Durringtonia*, and *Opercularia*) as successive sisters to a *Leptostigma* – *Pomax* clade (e.g., Figure 3c). When included, *Durringtonia* and *Opercularia* are sisters (Figure 4).

## AUTHOR CONTRIBUTIONS

**Olle Thureborn:** Conceptualization (equal); data curation (lead); formal analysis (lead); investigation (lead); validation (equal); visualization (equal); writing – original draft (lead); writing – review and editing (equal). **Niklas Wikström:** Conceptualization (equal); data curation (supporting); formal analysis (supporting); investigation (supporting); supervision (supporting); validation (equal); writing – original draft (supporting); writing – review and editing (equal). **Sylvain G. Razafimandimbison:** Conceptualization (equal); investigation (supporting); supervision (supporting); validation (equal); writing – original draft (supporting); writing – review and editing (equal). **Catarina Rydin:** Conceptualization (equal); funding acquisition (lead); investigation (supporting); project administration (lead); resources (lead); supervision (lead); validation (equal); visualization (equal); writing – original draft (supporting); writing – review and editing (equal).

## CONFLICT OF INTEREST STATEMENT

The authors declare that no conflicts of interest exist.

## Supporting information


Appendix S1.

## Data Availability

Raw sequence reads used in this study were deposited in the European Nucleotide Archive (ENA) under project PRJEB62527 (see Table S1 for sample‐specific accession numbers). The assembled sequences have been uploaded to GenBank (see Table S1 for accession numbers). Sequence alignments of molecular markers used in the study are available from the Dryad digital repository (doi: 10.5061/dryad.80gb5mkx4).
