# Supplementary material for: Phylogenomics and topological conflicts in the tribe Anthospermeae (Rubiaceae)
Source: Ecol Evol. 2024 Jan 25;14(1):e10868. doi: 10.1002/ece3.10868 (PMC10809029; doi:10.1002/ece3.10868)
Supplement: Supplementary file 1 — Appendix S1. [file ECE3-14-e10868-s001.pdf]

APPENDIX S1

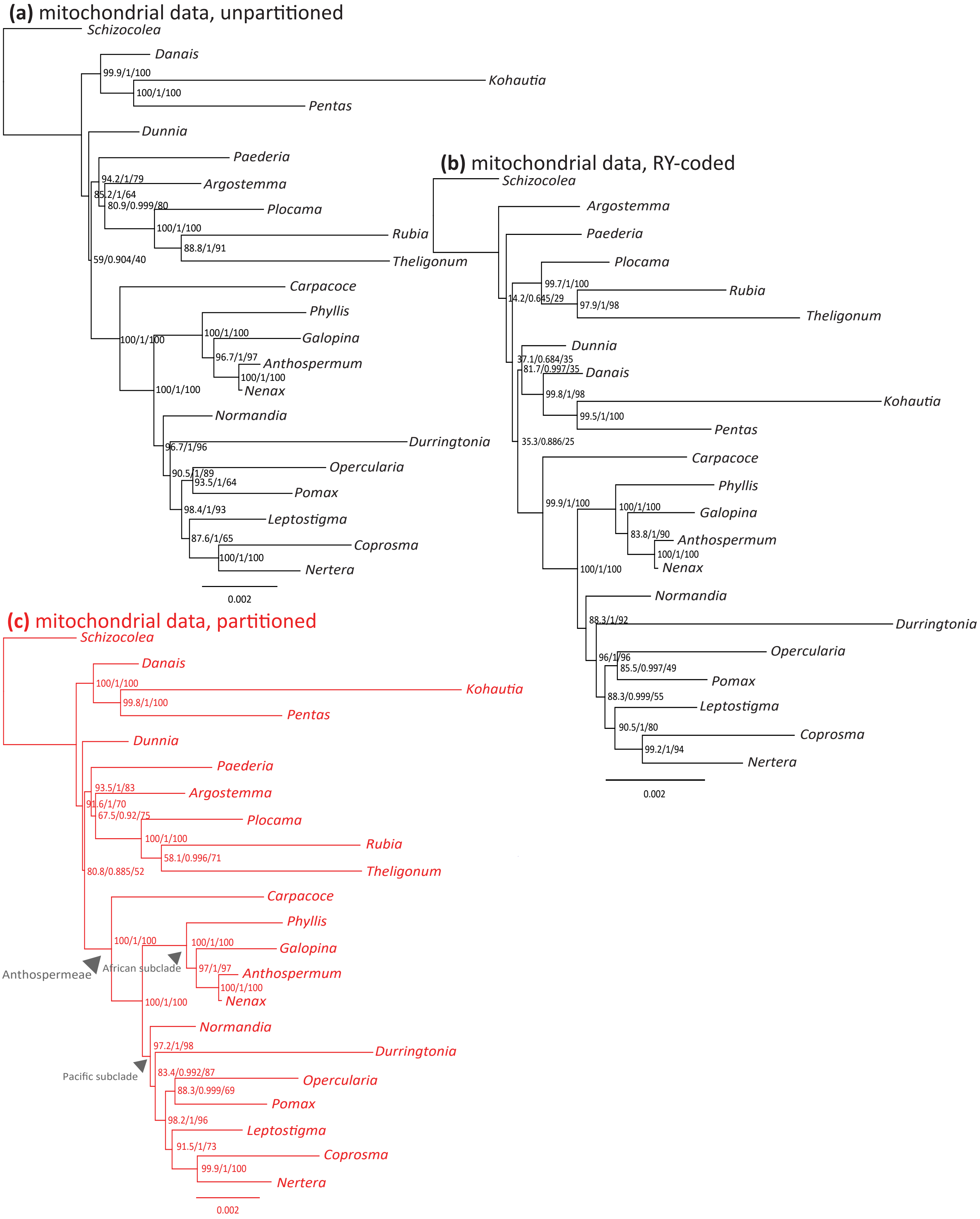

FIGURE S1 Phylogenetic results based on maximum likelihood analyses of mitochondrial data. (a) unpartitioned dataset, (b) RY-coded dataset, and for comparison (c) partitioned dataset (also presented in Figure 3). Values at nodes represent ultrafast bootstrap (UFboot), approximate Bayes (aBayes) and approximate likelihood ratio test (SH-aLRT) support values.

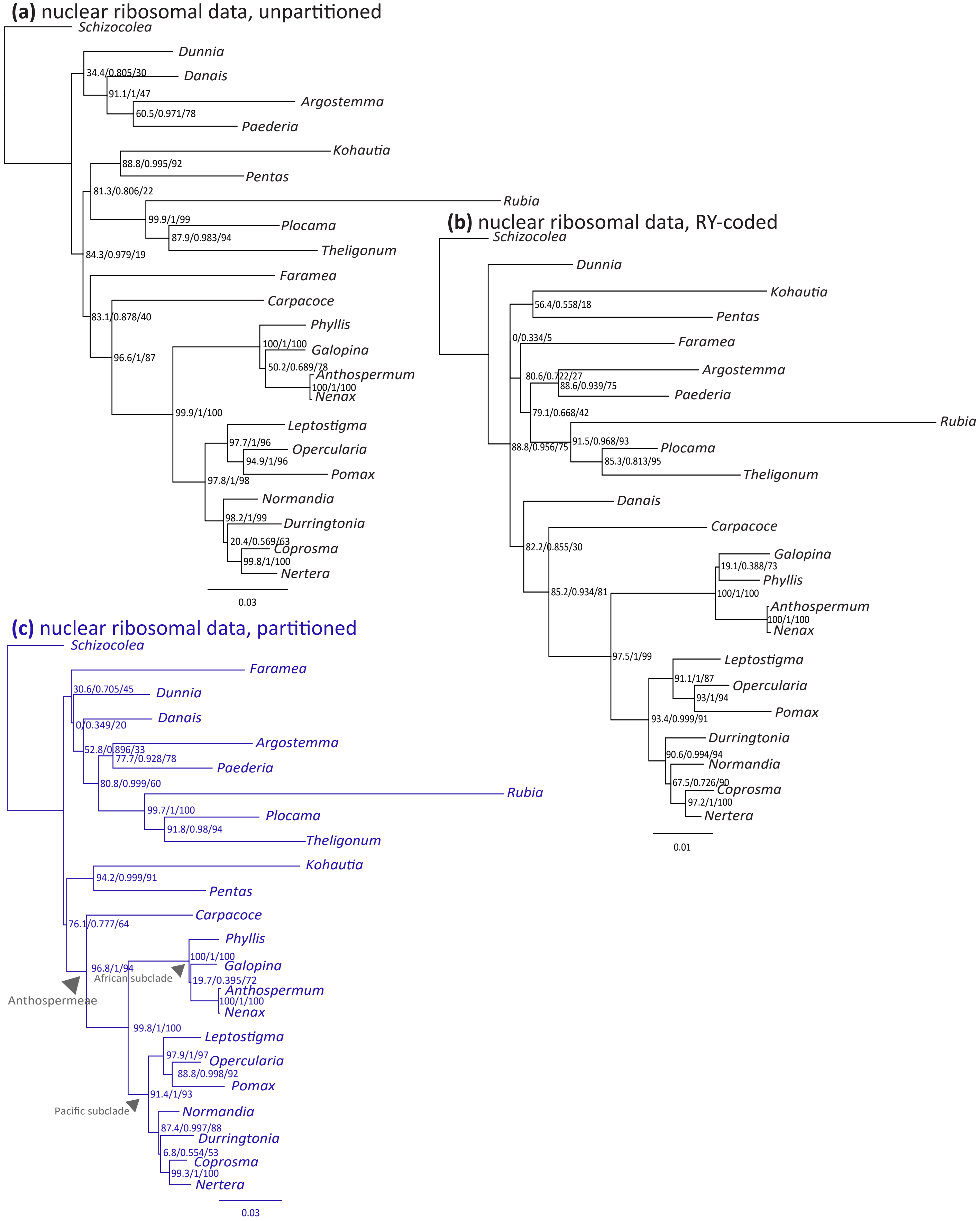

FIGURE S2 Phylogenetic results based on maximum likelihood analyses of nuclear ribosomal data. (a) unpartitioned dataset, (b) RY-coded dataset, and for comparison (c) partitioned dataset (also presented in Figure 3). Values at nodes represent ultrafast bootstrap (UFboot), approximate Bayes (aBayes) and approximate likelihood ratio test (SH-aLRT) support values.

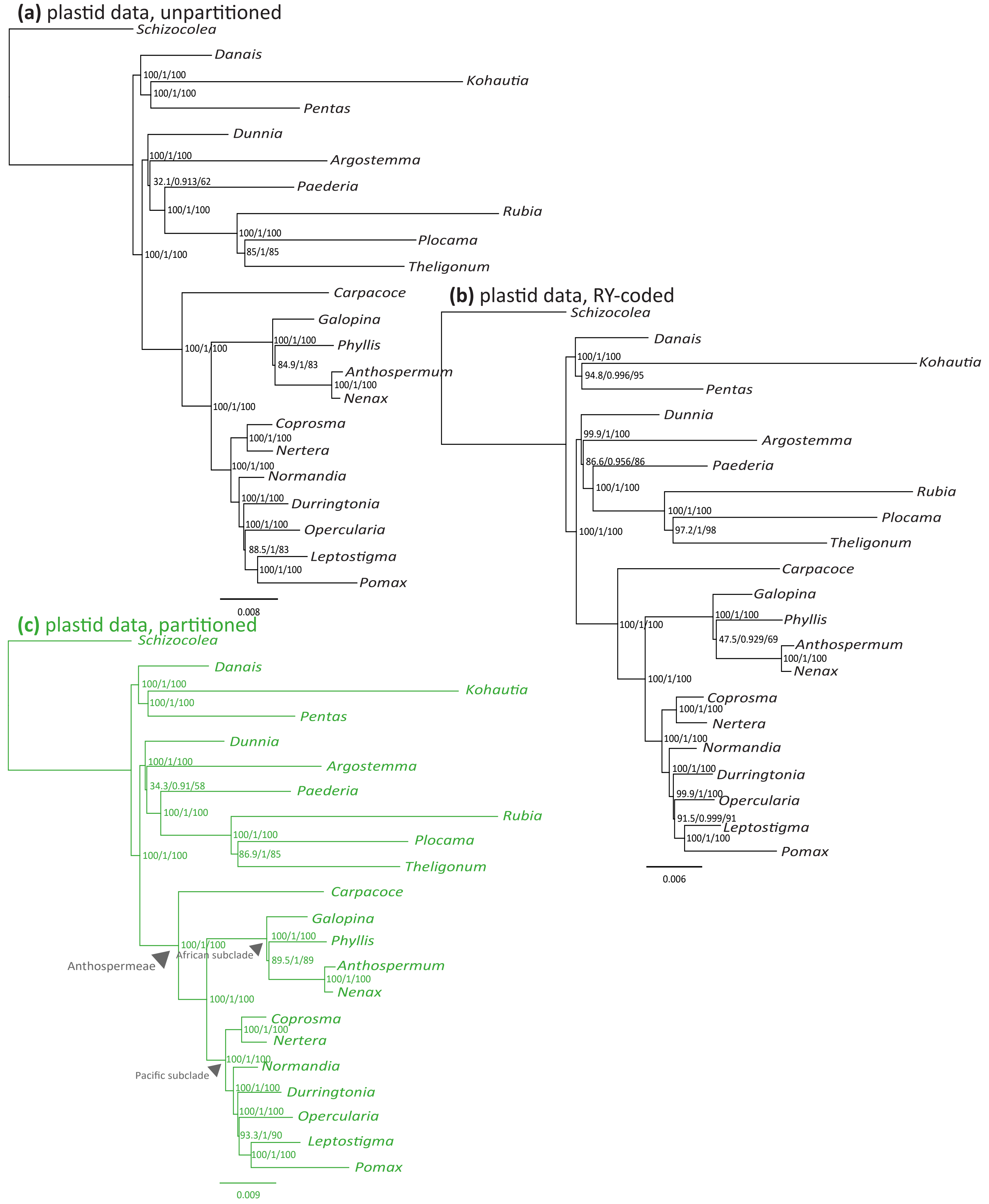

FIGURE S3 Phylogenetic results based on maximum likelihood analyses of plastid data (entire dataset). (a) unpartitioned dataset, (b) RY-coded dataset, and for comparison (c) partitioned dataset (also presented in Figure 3). Values at nodes represent ultrafast bootstrap (UFboot), approximate Bayes (aBayes) and approximate likelihood ratio test (SH-aLRT) support values.

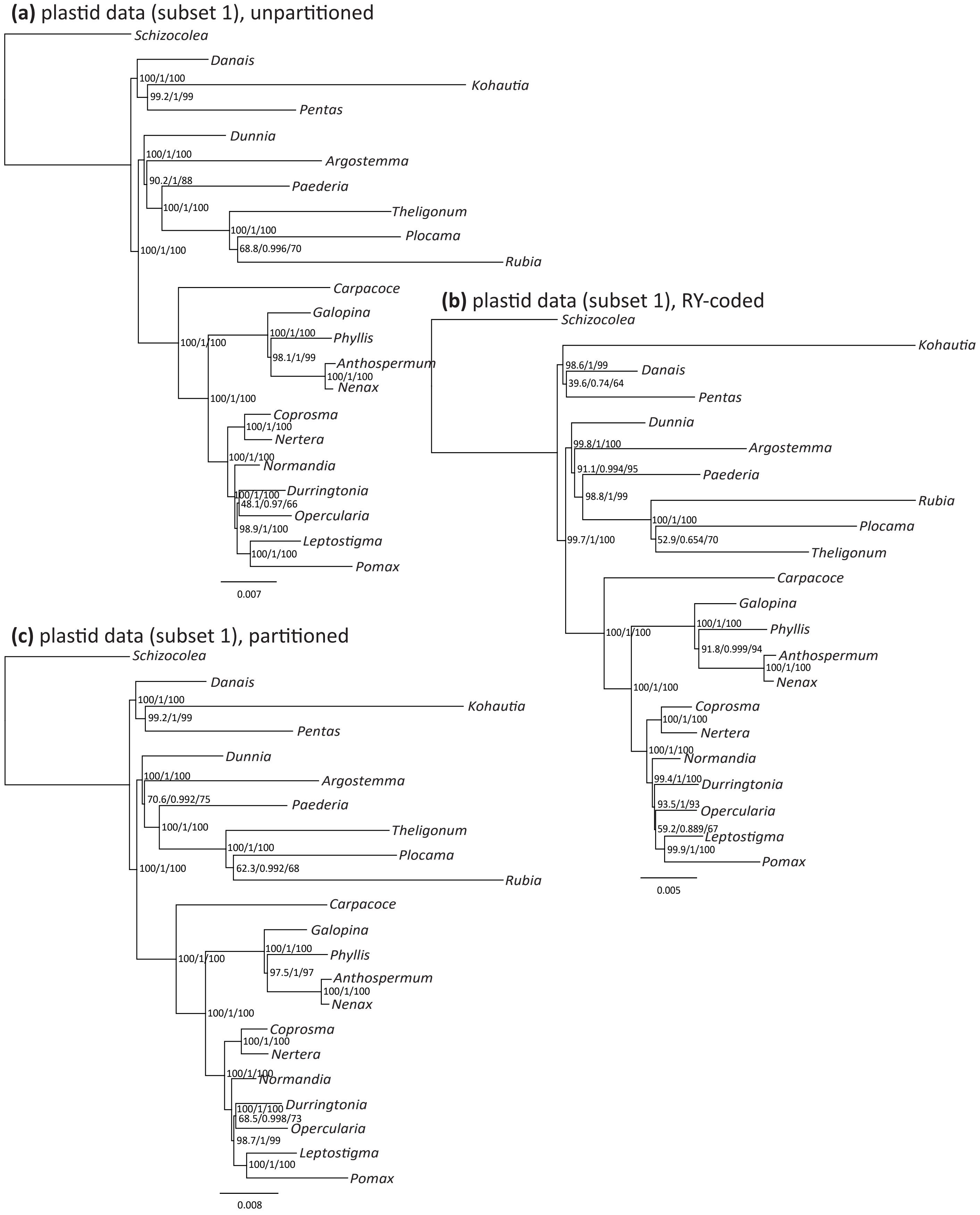

FIGURE S4 Phylogenetic results based on maximum likelihood analyses of plastid data (subset 1). (a) unpartitioned dataset, (b) RY-coded dataset, (c) partitioned dataset. Values at nodes represent ultrafast bootstrap (UFboot), approximate Bayes (aBayes) and approximate likelihood ratio test (SH-aLRT) support values.

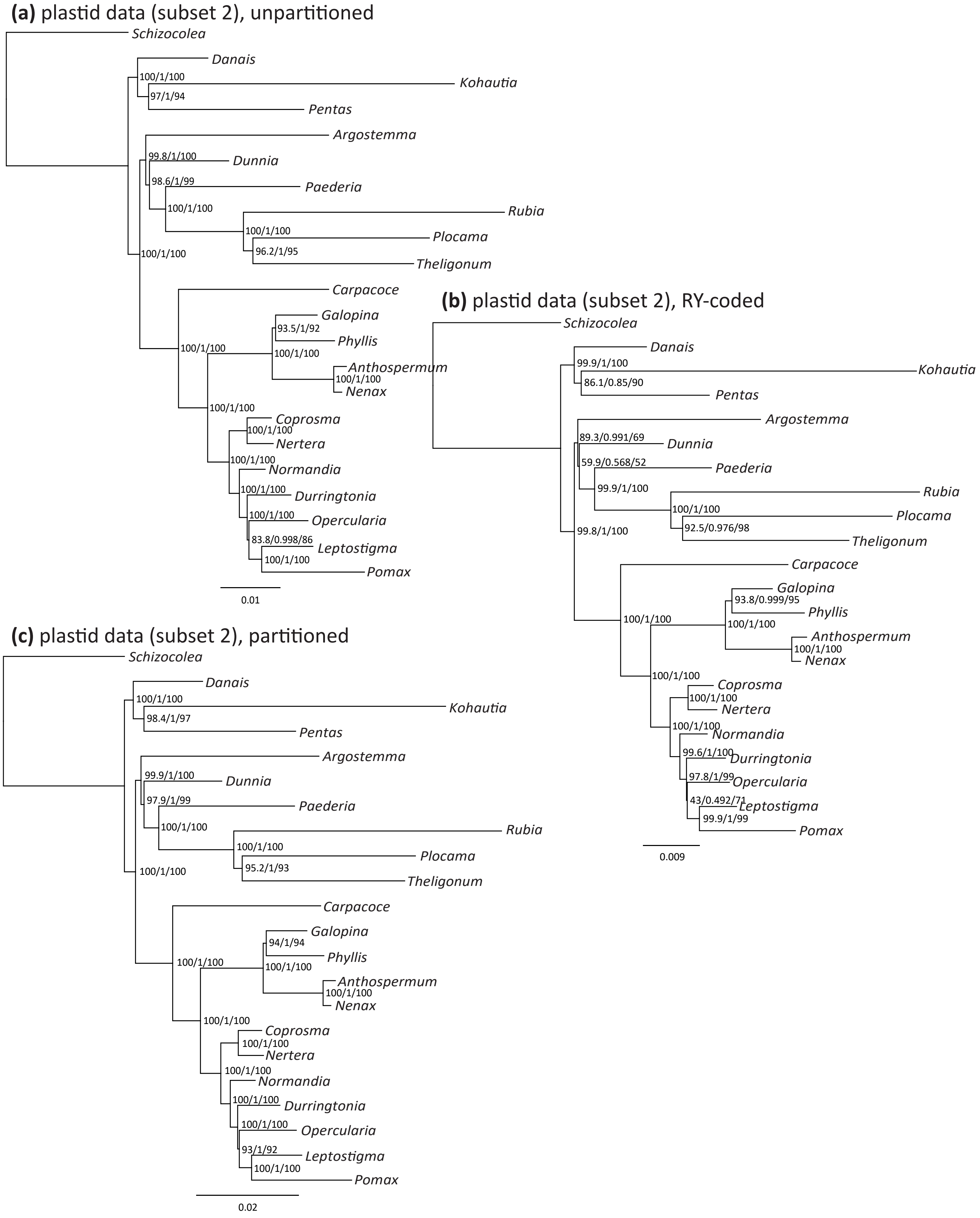

FIGURE S5 Phylogenetic results based on maximum likelihood analyses of plastid data (subset 2). (a) unpartitioned dataset, (b) RY-coded dataset, (c) partitioned dataset. Values at nodes represent ultrafast bootstrap (UFboot), approximate Bayes (aBayes) and approximate likelihood ratio test (SH-aLRT) support values.

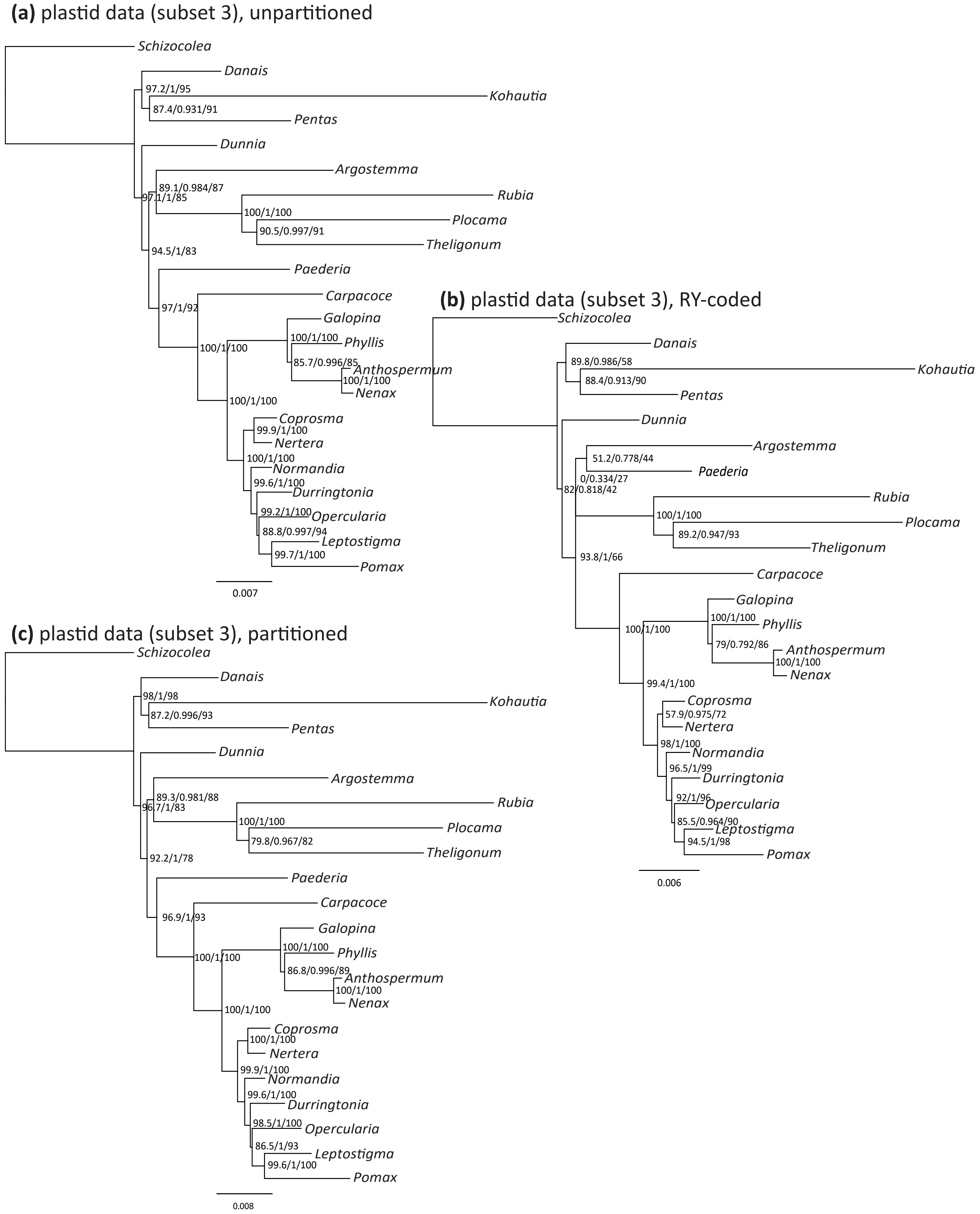

FIGURE S6 Phylogenetic results based on maximum likelihood analyses of plastid data (subset 3). (a) unpartitioned dataset, (b) RY-coded dataset, (c) partitioned dataset. Values at nodes represent ultrafast bootstrap (UFboot), approximate Bayes (aBayes) and approximate likelihood ratio test (SH-aLRT) support values.

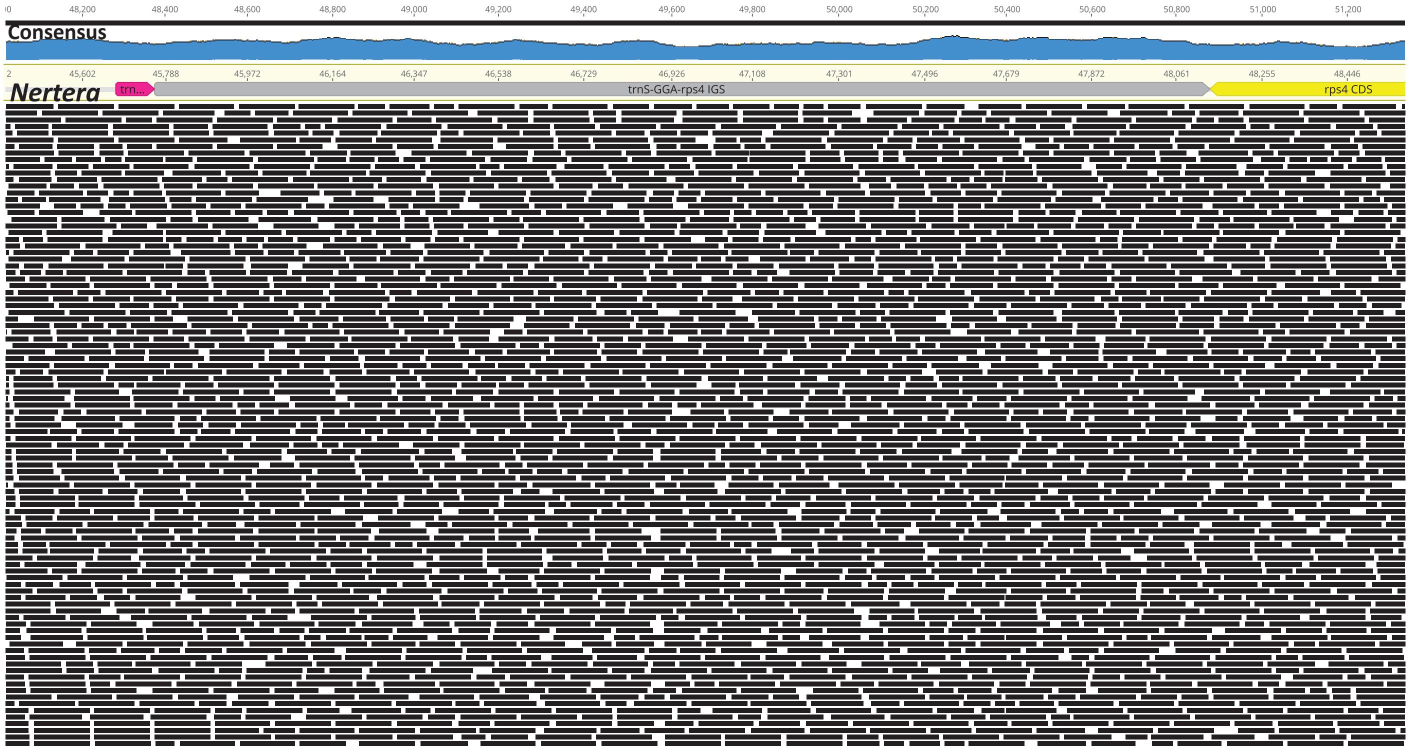

FIGURE S7 Sequence depth of the *trnS$_{GGA}$-rps4* region of *Nertera granadensis,* voucher: Chung & Anderberg 1348 (S).

TABLE S1

List of taxon names, DNA voucher information and accession numbers for sequences used.

Species	Voucher/Source	Collection year	Locality	ENA raw reads	Plastome	nrDNA cistron
Anthospermum aethiopicum L.	Bremer et al. 4363 (UPS)	2002	South Africa	ERR11475409	OR074081	OR074093
Carpacoce spermacocea Sond.	Bremer & Bremer 3708 (UPS)	1996	South Africa	ERR11475419	OR074082	OR074094
Coprosma rotundifolia A.Cunn.	Tibell NZ 65 (UPS)	1980	New Zealand	ERR11475416	OR074083	OR074095
Durringtonia paludosa R.J.F.Hend. & Guymer	Henderson et al. H 3044 (NSW)	1983	Australia	ERR11475415	OR074084	OR074096
Galopina circaeoides Thunb.	Bremer & Bremer 3797 (UPS)	1997	South Africa	ERR11475414	OR074092	OR074097
Leptostigma pilosum (Benth.) Fosberg	Asplund 7171 (UPS)	1939	Ecuador	ERR11475413	OR074085	OR074098
Nenax microphylla (Sond.) T.M.Salter	Hafström & Acock 1441 (S)	1938	South Africa	ERR11475408	OR074086	OR074099
Nertera granadensis Druce	Chung & Anderberg 1348 (S)	1999	Taiwan	ERR11475417	OR074087	OR074100
Normandia neocaledonica Hook.f.	Munzinger 532 (MO)	2001	New Caledonia	ERR11475418	OR074088	OR074101
Opercularia varia Hook.f.	Karunajeewa 832 (S)	2013	Australia	ERR11475411	OR074089	OR074102
Phyllis viscosa Christ	Santesson 26911 (S)	1976	Canary Islands, Teneriffe	ERR11475412	OR074090	OR074103
Pomax umbellata Sol. ex Gaertn.	Bremer & Bremer 3918 (UPS)	1998	Australia	ERR11475410	OR074091	OR074104
Argostemma yappii King	Rydin et al. (2017)				KY378693	MK607894
Danais xanthorrhoea (K.Schum.) Bremek.	Rydin et al. (2017)				KY378686	MK607903
Dunnia sinensis Tutch.	Rydin et al. (2017)				KY378692	MK607905
Faramea multiflora A.Rich. ex DC.	Rydin et al. (2017)					MK607906
Kohautia caespitosa Schnizl.	Rydin et al. (2017)				KY378684	MK607918
Paederia foetida L.	Rydin et al. (2017)				KY378691	MK607927
Pentas lanceolata (Forssk.) Deflers	Rydin et al. (2017)				KY378685	MK607931
Plocama pendula Aiton	Rydin et al. (2017)				KY378690	MK607932
Rubia horrida (Thunb.) Puff	Rydin et al. (2017)				KY378689	MK607939
Schizocolea linderi (Hutch. & Dalziel) Bremek.	Rydin et al. (2017)				KY378700	MK607941
Theligonum cynocrambe L.	Rydin et al. (2017)				KY378688	MK607946

Newly sequenced taxa in bold face

TABLE S1

List of taxon names, DNA voucher information and accession numbers for sequences used.

Species	Voucher/Source	*atp1*	*atp4*	*atp6*	*atp8*	*atp9*	*ccmB*	*ccmC*
Anthospermum aethiopicum L.	Bremer et al. 4363 (UPS)	OR794561	OR794573	OR794585	OR794597	OR794609	OR794621	OR794789
Carpacoce spermacocea Sond.	Bremer & Bremer 3708 (UPS)	OR794562	OR794574	OR794586	OR794598	OR794610	OR794622	OR794790
Coprosma rotundifolia A.Cunn.	Tibell NZ 65 (UPS)	OR794563	OR794575	OR794587	OR794599	OR794611	OR794623	OR794791
Durringtonia paludosa R.J.F.Hend. & Guymer	Henderson et al. H 3044 (NSW)	OR794564	OR794576	OR794588	OR794600	OR794612	OR794624	OR794792
Galopina circaeoides Thunb.	Bremer & Bremer 3797 (UPS)	OR794565	OR794577	OR794589	OR794601	OR794613	OR794625	OR794793
Leptostigma pilosum (Benth.) Fosberg	Asplund 7171 (UPS)	OR794566	OR794578	OR794590	OR794602	OR794614	OR794626	OR794794
Nenax microphylla (Sond.) T.M.Salter	Hafström & Acock 1441 (S)	OR794567	OR794579	OR794591	OR794603	OR794615	OR794627	OR794795
Nertera granadensis Druce	Chung & Anderberg 1348 (S)	OR794568	OR794580	OR794592	OR794604	OR794616	OR794628	OR794796
Normandia neocaledonica Hook.f.	Munzinger 532 (MO)	OR794569	OR794581	OR794593	OR794605	OR794617	OR794629	OR794797
Opercularia varia Hook.f.	Karunajeewa 832 (S)	OR794570	OR794582	OR794594	OR794606	OR794618	OR794630	OR794798
Phyllis viscosa Christ	Santesson 26911 (S)	OR794571	OR794583	OR794595	OR794607	OR794619	OR794631	OR794799
Pomax umbellata Sol. ex Gaertn.	Bremer & Bremer 3918 (UPS)	OR794572	OR794584	OR794596	OR794608	OR794620	OR794632	OR794800
Argostemma yappii King	Rydin et al. (2017)	KY638152	KY637402	KY638787	KY638268	KY638498	KY638094	KY639019
Danais xanthorrhoea (K.Schum.) Bremek.	Rydin et al. (2017)	KY638147	KY637397	KY638782	KY638263	KY638494	KY638089	KY639014
Dunnia sinensis Tutch.	Rydin et al. (2017)	KY638161	KY637411	KY638796	KY638277	KY638506	KY638103	KY639028
Faramea multiflora A.Rich. ex DC.	Rydin et al. (2017)							
Kohautia caespitosa Schnizl.	Rydin et al. (2017)	KY638149	KY637399	KY638784	KY638265	KY638496	KY638091	KY639016
Paederia foetida L.	Rydin et al. (2017)	KY638174	KY637424	KY638809	KY638290	KY638519	KY638116	KY639041
Pentas lanceolata (Forssk.) Deflers	Rydin et al. (2017)	KY638148	KY637398	KY638783	KY638264	KY638495	KY638090	KY639015
Plocama pendula Aiton	Rydin et al. (2017)	KY638177	KY637427	KY638813	KY638293	KY638522	KY638119	KY639044
Rubia horrida (Thunb.) Puff	Rydin et al. (2017)	KY638182	KY637432	KY638817	KY638298	KY638527	KY638124	KY639049
Schizocolea linderi (Hutch. & Dalziel) Bremek.	Rydin et al. (2017)	KY638140	KY637390	KY638775	KY638256	KY638487	KY638082	KY639007
Theligonum cynocrambe L.	Rydin et al. (2017)	KY638188	KY637438	KY638823	KY638304	KY638533	KY638130	KY639055

Newly sequenced taxa in bold face

TABLE S1

List of taxon names, DNA voucher information and accession numbers for sequences used.

Species	Voucher/Source	*ccmFc*	*ccmFn*	*cob*	*cox1*	*cox2*	*cox3*	*matR*
Anthospermum aethiopicum L.	Bremer et al. 4363 (UPS)	OR794825	OR794633	OR794645	OR794657	OR794837	OR794669	OR794681
Carpacoce spermacocea Sond.	Bremer & Bremer 3708 (UPS)	OR794826	OR794634	OR794646	OR794658	OR794838	OR794670	OR794682
Coprosma rotundifolia A.Cunn.	Tibell NZ 65 (UPS)	OR794827	OR794635	OR794647	OR794659	OR794839	OR794671	OR794683
Durringtonia paludosa R.J.F.Hend. & Guymer	Henderson et al. H 3044 (NSW)	OR794828	OR794636	OR794648	OR794660	OR794840	OR794672	OR794684
Galopina circaeoides Thunb.	Bremer & Bremer 3797 (UPS)	OR794829	OR794637	OR794649	OR794661	OR794841	OR794673	OR794685
Leptostigma pilosum (Benth.) Fosberg	Asplund 7171 (UPS)	OR794830	OR794638	OR794650	OR794662	OR794842	OR794674	OR794686
Nenax microphylla (Sond.) T.M.Salter	Hafström & Acock 1441 (S)	OR794831	OR794639	OR794651	OR794663	OR794843	OR794675	OR794687
Nertera granadensis Druce	Chung & Anderberg 1348 (S)	OR794832	OR794640	OR794652	OR794664	OR794844	OR794676	OR794688
Normandia neocaledonica Hook.f.	Munzinger 532 (MO)	OR794833	OR794641	OR794653	OR794665	OR794845	OR794677	OR794689
Opercularia varia Hook.f.	Karunajeewa 832 (S)	OR794834	OR794642	OR794654	OR794666	OR794846	OR794678	OR794690
Phyllis viscosa Christ	Santesson 26911 (S)	OR794835	OR794643	OR794655	OR794667	OR794847	OR794679	OR794691
Pomax umbellata Sol. ex Gaertn.	Bremer & Bremer 3918 (UPS)	OR794836	OR794644	OR794656	OR794668	OR794848	OR794680	OR794692
Argostemma yappii King	Rydin et al. (2017)	KY637576	KY638903	KY638961	KY637920	KY637634	KY638210	KY6375I8
Danais xanthorrhoea (K.Schum.) Bremek.	Rydin et al. (2017)	KY637571	KY638898	KY638956	KY637915	KY637629	KY638205	KY637513
Dunnia sinensis Tutch.	Rydin et al. (2017)	KY637585	KY638912	KY638970	KY637929	KY637643	KY638219	KY637527
Faramea multiflora A.Rich. ex DC.	Rydin et al. (2017)							
Kohautia caespitosa Schnizl.	Rydin et al. (2017)	KY637573	KY638900	KY638958	KY637917	KY637631	KY638207	KY637515
Paederia foetida L.	Rydin et al. (2017)	KY637598	KY638925	KY638983	KY637942	KY637656	KY638232	KY637540
Pentas lanceolata (Forssk.) Deflers	Rydin et al. (2017)	KY637572	KY638899	KY638957	KY637916	KY637630	KY638206	KY637514
Plocama pendula Aiton	Rydin et al. (2017)	KY637601	KY638928	KY638986	KY637945	KY637659	KY638235	KY637543
Rubia horrida (Thunb.) Puff	Rydin et al. (2017)	KY637606	KY638933	KY638991	KY637950	KY637664	KY638240	KY637548
Schizocolea linderi (Hutch. & Dalziel) Bremek.	Rydin et al. (2017)	KY637564	KY638891	KY638949	KY637908	KY637622	KY638198	KY637506
Theligonum cynocrambe L.	Rydin et al. (2017)	KY637612	KY638939	KY638997	KY637956	KY637670	KY638246	KY637554

Newly sequenced taxa in bold face

TABLE S1

List of taxon names, DNA voucher information and accession numbers for sequences used.

Species	Voucher/Source	*mttB*	*nad1 exon 1*	*nad1 exon 2 to 3*	*nad1 exon 5*	*nad2 exon 1 to 2*	*nad2 exon 3 to 5*	*nad3*
Anthospermum aethiopicum L.	Bremer et al. 4363 (UPS)	OR794801	OR794873	OR794885	OR794897	OR794909	OR794921	OR794693
Carpacoce spermacocea Sond.	Bremer & Bremer 3708 (UPS)	OR794802	OR794874	OR794886	OR794898	OR794910	OR794922	OR794694
Coprosma rotundifolia A.Cunn.	Tibell NZ 65 (UPS)	OR794803	OR794875	OR794887	OR794899	OR794911	OR794923	OR794695
Durringtonia paludosa R.J.F.Hend. & Guymer	Henderson et al. H 3044 (NSW)	OR794804	OR794876	OR794888	OR794900	OR794912	OR794924	OR794696
Galopina circaeoides Thunb.	Bremer & Bremer 3797 (UPS)	OR794805	OR794877	OR794889	OR794901	OR794913	OR794925	OR794697
Leptostigma pilosum (Benth.) Fosberg	Asplund 7171 (UPS)	OR794806	OR794878	OR794890	OR794902	OR794914	OR794926	OR794698
Nenax microphylla (Sond.) T.M.Salter	Hafström & Acock 1441 (S)	OR794807	OR794879	OR794891	OR794903	OR794915	OR794927	OR794699
Nertera granadensis Druce	Chung & Anderberg 1348 (S)	OR794808	OR794880	OR794892	OR794904	OR794916	OR794928	OR794700
Normandia neocaledonica Hook.f.	Munzinger 532 (MO)	OR794809	OR794881	OR794893	OR794905	OR794917	OR794929	OR794701
Opercularia varia Hook.f.	Karunajeewa 832 (S)	OR794810	OR794882	OR794894	OR794906	OR794918	OR794930	OR794702
Phyllis viscosa Christ	Santesson 26911 (S)	OR794811	OR794883	OR794895	OR794907	OR794919	OR794931	OR794703
Pomax umbellata Sol. ex Gaertn.	Bremer & Bremer 3918 (UPS)	OR794812	OR794884	OR794896	OR794908	OR794920	OR794932	OR794704
Argostemma yappii King	Rydin et al. (2017)	KY639135	KY637746	KY637804	KY637862	KY638555	KY638613	KY637228
Danais xanthorrhoea (K.Schum.) Bremek.	Rydin et al. (2017)	KY639130	KY637741	KY637799	KY637857	KY638550	KY638608	KY637223
Dunnia sinensis Tutch.	Rydin et al. (2017)	KY639144	KY637755	KY637813	KY637871	KY638564	KY638622	KY637237
Faramea multiflora A.Rich. ex DC.	Rydin et al. (2017)							
Kohautia caespitosa Schnizl.	Rydin et al. (2017)	KY639132	KY637743	KY637801	KY637859	KY638552	KY638610	KY637225
Paederia foetida L.	Rydin et al. (2017)	KY639157	KY637768	KY637826	KY637884	KY638577	KY638635	KY637250
Pentas lanceolata (Forssk.) Deflers	Rydin et al. (2017)	KY639131	KY637742	KY637800	KY637858	KY638551	KY638609	KY637224
Plocama pendula Aiton	Rydin et al. (2017)	KY639160	KY637771	KY637829	KY637887	KY638580	KY638638	KY637253
Rubia horrida (Thunb.) Puff	Rydin et al. (2017)	KY639165	KY637776	KY637834	KY637892	KY638585	KY638643	KY637258
Schizocolea linderi (Hutch. & Dalziel) Bremek.	Rydin et al. (2017)	KY639123	KY637734	KY637792	KY637850	KY638543	KY638601	KY637216
Theligonum cynocrambe L.	Rydin et al. (2017)	KY639171	KY637782	KY637840	KY637898	KY638591	KY638649	KY637264

Newly sequenced taxa in bold face

TABLE S1

List of taxon names, DNA voucher information and accession numbers for sequences used.

Species	Voucher/Source	*nad4*	*nad4L*	*nad5 exon 1 to 2*	*nad5 exon 4 to 5*	*nad6*	*nad7*	*nad9*
Anthospermum aethiopicum L.	Bremer et al. 4363 (UPS)	OR794933	OR794705	OR794945	OR794957	OR794717	OR794969	OR794729
Carpacoce spermacocea Sond.	Bremer & Bremer 3708 (UPS)	OR794934	OR794706	OR794946	OR794958	OR794718	OR794970	OR794730
Coprosma rotundifolia A.Cunn.	Tibell NZ 65 (UPS)	OR794935	OR794707	OR794947	OR794959	OR794719	OR794971	OR794731
Durringtonia paludosa R.J.F.Hend. & Guymer	Henderson et al. H 3044 (NSW)	OR794936	OR794708	OR794948	OR794960	OR794720	OR794972	OR794732
Galopina circaeoides Thunb.	Bremer & Bremer 3797 (UPS)	OR794937	OR794709	OR794949	OR794961	OR794721	OR794973	OR794733
Leptostigma pilosum (Benth.) Fosberg	Asplund 7171 (UPS)	OR794938	OR794710	OR794950	OR794962	OR794722	OR794974	OR794734
Nenax microphylla (Sond.) T.M.Salter	Hafström & Acock 1441 (S)	OR794939	OR794711	OR794951	OR794963	OR794723	OR794975	OR794735
Nertera granadensis Druce	Chung & Anderberg 1348 (S)	OR794940	OR794712	OR794952	OR794964	OR794724	OR794976	OR794736
Normandia neocaledonica Hook.f.	Munzinger 532 (MO)	OR794941	OR794713	OR794953	OR794965	OR794725	OR794977	OR794737
Opercularia varia Hook.f.	Karunajeewa 832 (S)	OR794942	OR794714	OR794954	OR794966	OR794726	OR794978	OR794738
Phyllis viscosa Christ	Santesson 26911 (S)	OR794943	OR794715	OR794955	OR794967	OR794727	OR794979	OR794739
Pomax umbellata Sol. ex Gaertn.	Bremer & Bremer 3918 (UPS)	OR794944	OR794716	OR794956	OR794968	OR794728	OR794980	OR794740
Argostemma yappii King	Rydin et al. (2017)	KY492154	KY637344	KY638383	KY638441	KY638845		KY638326
Danais xanthorrhoea (K.Schum.) Bremek.	Rydin et al. (2017)	KY492149	KY637339	KY638378	KY638436	KY638840	KY638031	KY638321
Dunnia sinensis Tutch.	Rydin et al. (2017)	KY492163	KY637353	KY638392	KY638450	KY638854	KY638045	KY638335
Faramea multiflora A.Rich. ex DC.	Rydin et al. (2017)							
Kohautia caespitosa Schnizl.	Rydin et al. (2017)	KY492151	KY637341	KY638380	KY638438	KY638842	KY638033	KY638323
Paederia foetida L.	Rydin et al. (2017)	KY492176	KY637366	KY638405	KY638463	KY638867	KY638058	KY638348
Pentas lanceolata (Forssk.) Deflers	Rydin et al. (2017)	KY492150	KY637340	KY638379	KY638437	KY638841	KY638032	KY638322
Plocama pendula Aiton	Rydin et al. (2017)	KY492179	KY637369	KY638408	KY638466	KY638870	KY638061	KY638351
Rubia horrida (Thunb.) Puff	Rydin et al. (2017)	KY492184	KY637374	KY638413	KY638471	KY638875	KY638066	KY638356
Schizocolea linderi (Hutch. & Dalziel) Bremek.	Rydin et al. (2017)	KY492142	KY637332	KY638371	KY638429	KY638833	KY638024	KY638314
Theligonum cynocrambe L.	Rydin et al. (2017)	KY492190	KY637380	KY638419	KY638477	KY638881	KY638072	KY638361

Newly sequenced taxa in bold face

TABLE S1

List of taxon names, DNA voucher information and accession numbers for sequences used.

Species	Voucher/Source	*rpl5*	*rps1*	*rps3*	*rps4*	*rps10*	*rps12*	*rps13*
Anthospermum aethiopicum L.	Bremer et al. 4363 (UPS)	OR794813	OR794741	OR794849	OR794753	OR794861	OR794765	OR794777
Carpacoce spermacocea Sond.	Bremer & Bremer 3708 (UPS)	OR794814	OR794742	OR794850	OR794754	OR794862	OR794766	OR794778
Coprosma rotundifolia A.Cunn.	Tibell NZ 65 (UPS)	OR794815	OR794743	OR794851	OR794755	OR794863	OR794767	OR794779
Durringtonia paludosa R.J.F.Hend. & Guymer	Henderson et al. H 3044 (NSW)	OR794816	OR794744	OR794852	OR794756	OR794864	OR794768	OR794780
Galopina circaeoides Thunb.	Bremer & Bremer 3797 (UPS)	OR794817	OR794745	OR794853	OR794757	OR794865	OR794769	OR794781
Leptostigma pilosum (Benth.) Fosberg	Asplund 7171 (UPS)	OR794818	OR794746	OR794854	OR794758	OR794866	OR794770	OR794782
Nenax microphylla (Sond.) T.M.Salter	Hafström & Acock 1441 (S)	OR794819	OR794747	OR794855	OR794759	OR794867	OR794771	OR794783
Nertera granadensis Druce	Chung & Anderberg 1348 (S)	OR794820	OR794748	OR794856	OR794760	OR794868	OR794772	OR794784
Normandia neocaledonica Hook.f.	Munzinger 532 (MO)	OR794821	OR794749	OR794857	OR794761	OR794869	OR794773	OR794785
Opercularia varia Hook.f.	Karunajeewa 832 (S)	OR794822	OR794750	OR794858	OR794762	OR794870	OR794774	OR794786
Phyllis viscosa Christ	Santesson 26911 (S)	OR794823	OR794751	OR794859	OR794763	OR794871	OR794775	OR794787
Pomax umbellata Sol. ex Gaertn.	Bremer & Bremer 3918 (UPS)	OR794824	OR794752	OR794860	OR794764	OR794872	OR794776	OR794788
Argostemma yappii King	Rydin et al. (2017)	KY639077	KY637692	KY639233	KY637460	KY492096	KY637286	KY637978
Danais xanthorrhoea (K.Schum.) Bremek.	Rydin et al. (2017)	KY639072	KY637687	KY639228	KY637455	KY492092	KY637281	KY637973
Dunnia sinensis Tutch.	Rydin et al. (2017)	KY639086	KY637699	KY639242	KY637469	KY492105	KY637295	KY637987
Faramea multiflora A.Rich. ex DC.	Rydin et al. (2017)							
Kohautia caespitosa Schnizl.	Rydin et al. (2017)	KY639074	KY637689	KY639230	KY637457		KY637283	KY637975
Paederia foetida L.	Rydin et al. (2017)	KY639099	KY637710	KY639255	KY637482	KY492118	KY637308	KY638000
Pentas lanceolata (Forssk.) Deflers	Rydin et al. (2017)	KY639073	KY637688	KY639229	KY637456	KY492093	KY637282	KY637974
Plocama pendula Aiton	Rydin et al. (2017)	KY639102	KY637713	KY639258	KY637485	KY492121	KY637311	KY638003
Rubia horrida (Thunb.) Puff	Rydin et al. (2017)	KY639107	KY637718	KY639263	KY637490	KY492126	KY637316	KY638008
Schizocolea linderi (Hutch. & Dalziel) Bremek.	Rydin et al. (2017)	KY639065	KY637680	KY639221	KY637448	KY492085	KY637274	KY637966
Theligonum cynocrambe L.	Rydin et al. (2017)	KY639113	KY637724	KY639269	KY637496	KY492132	KY637322	KY638014

Newly sequenced taxa in bold face

TABLE S2

Loci/markers with identified putative hairpin regions.

Locus/Marker	Genomic compartment	Data type
ccmFc intron	mitogenome	intron
nad1 intron 2	mitogenome	intron
rps3 intron	mitogenome	intron
matR	mitogenome	PCG
ccsA-ndhD	plastome	IGS
petB-petD	plastome	IGS
petD-rpoA	plastome	IGS
psbC-trnS ^{UGA}	plastome	IGS
rpoC2-rpoC1	plastome	IGS
trnK-rps16	plastome	IGS
trnR-trnN	plastome	IGS
trnW-trnP	plastome	IGS
ycf2-trnL ^{CAA}	plastome	IGS
petB intron	plastome	intron
petD intron	plastome	intron
trnL intron	plastome	intron
ycf2	plastome	PCG

TABLE S3

Assembly statistics. Taxon with fully assembled plastome is indicated in grey.

Taxon	Plastome length	Plastome*	*trnS-rps4* IGS length	*trnS-rps4* IGS*	*trnS-GCU-petA**	*rps4-psbJ**	Mitochondrial genes length (bp)	Mitochondrial genes*	Nuclear ribosomal cistron length	Nuclear ribsomal cistron*
Anthospermum	153 839	430.2	267	461.0	NA	NA	50 913	66.5	6 320	1 865.1
Carpacoce	155 631	487.8	308	309.1	NA	NA	51 259	66.3	7 172	581.1
Coprosma	155 707	126.5	NA	NA	107.1	121.0	52 855	19.3	7 218	1 105.0
Durringtonia	156 338	499.6	1 936	560.6	NA	NA	54 075	59.8	7 259	1 159.9
Galopina	154 371	423.7	302	419.1	NA	NA	51 158	88.0	7 570	473.3
Leptostigma	155 359	151.5	1 133	132.5	NA	NA	51 148	29.0	6 867	515.7
Nenax	153 810	222.0	267	238.7	NA	NA	51 014	58.1	6 652	606.9
Nertera	156 932	285.9	2 374	297.5	NA	NA	51 661	56.9	8 770	691.5
Normandia	156 049	797.5	1 955	872.0	NA	NA	51 801	244.6	7 125	2 630.1
Opercularia	156 016	757.4	1 896	834.7	NA	NA	61 205	203.7	7 734	1 739.7
Phyllis	153 709	1 444.5	290	1 444.2	NA	NA	51 448	158.5	6 848	567.8
Pomax	155 494	383.6	1 118	338.8	NA	NA	52 052	61.5	7 245	856.8

*average coverage

TABLE S4

Subsets of plastid data, identified using the hierarchical likelihood ratio test implemented in software Concaterpillar 1.8a.

Locus/Marker	Data type	Plastid subset	Locus/Marker	Data type	Plastid subset	Locus/Marker	Data type	Plastid subset
accD	PCG	1	trnI-trnA	IGS	1	rpl32-trnL^{UAG}	IGS	2
atpA	PCG	1	trnK-rps16	IGS	1	rpl36-rps8 incl infA	IGS	2
atpF	PCG	1	trnL-trnF^{GAA}	IGS	1	rps11-rpl36	IGS	2
atpI	PCG	1	trnQ-psbK	IGS	1	rps12-trnV^{GAC}	IGS	2
ccsA	PCG	1	trnR-trnN	IGS	1	rps14-psaB	IGS	2
cemA	PCG	1	trnR^{UCU}-atpA	IGS	1	rps16-trnQ	IGS	2
matK	PCG	1	trnS^{UGA}-psbZ	IGS	1	rps2-rpoC2	IGS	2
ndhA	PCG	1	trnT^{GGU}-psbD	IGS	1	rps7-rps12	IGS	2
ndhB	PCG	1	trnV-23S	IGS	1	trnD^{GUC}-trnY^{GUA}	IGS	2
ndhE	PCG	1	ycf2-trnL^{CAA}	IGS	1	trnE^{UUC}-trnT^{GGU}	IGS	2
ndhG	PCG	1	ycf3-trnS^{GGA}	IGS	1	trnG^{UCC}-trnfM^{CAU}	IGS	2
ndhK	PCG	1	atpF	intron	1	trnH^{GUG}-psbA	IGS	2
petA	PCG	1	clpPi2	intron	1	trnL^{CAA}-ndhB	IGS	2
petL	PCG	1	petB	intron	1	trnL^{UAG}-ccsA	IGS	2
psaA	PCG	1	petD	intron	1	trnN-ycf1	IGS	2
psbB	PCG	1	rps16	intron	1	trnP-psaJ	IGS	2
psbC	PCG	1	trnA	intron	1	trnS^{GCU}-trnG^{GCC}	IGS	2
psbE	PCG	1	trnK5	intron	1	trnT^{UGU}-trnL^{UAA}	IGS	2
psbI	PCG	1	trnV	intron	1	trnV^{CAU}-atpE	IGS	2
psbK	PCG	1	ycf3i2	intron	1	trnV^{GAC}-16SrRNA	IGS	2
psbN	PCG	1	tRNAs_30	tRNA	1	trnV^{UAC}-trnM^{CAU}	IGS	2
rpl16	PCG	1	atpB	PCG	2	trnW-trnP	IGS	2
rpl2	PCG	1	atpE	PCG	2	trnY^{GUA}-trnE^{UUC}	IGS	2
rpl20	PCG	1	atpH	PCG	2	ycf4-cemA	IGS	2
rpl23	PCG	1	ndhC	PCG	2	clpPi1	intron	2
rpl32	PCG	1	ndhD	PCG	2	ndhA	intron	2
rpoA	PCG	1	ndhH	PCG	2	rpl16	intron	2
rpoB	PCG	1	ndhI	PCG	2	rpl2	intron	2
rpoC2	PCG	1	ndhJ	PCG	2	rpoC1	intron	2
rps12	PCG	1	petN	PCG	2	trnG^{GCC}	intron	2
rps14	PCG	1	psaB	PCG	2	trnK3	intron	2
rps19	PCG	1	psaJ	PCG	2	ycf3i1	intron	2
rps4	PCG	1	psbA	PCG	2	clpP	PCG	3
rps7	PCG	1	psbD	PCG	2	ndhF	PCG	3
rps8	PCG	1	psbM	PCG	2	petB	PCG	3
ycf1	PCG	1	psbT	PCG	2	petD	PCG	3
ycf2	PCG	1	psbZ	PCG	2	psaC	PCG	3
ycf3	PCG	1	rbcL	PCG	2	psbH	PCG	3
atpF-atpH	IGS	1	rpl14	PCG	2	psbJ	PCG	3
atpH-atpI	IGS	1	rpl22	PCG	2	rps18	PCG	3
ccsA-ndhD	IGS	1	rpoC1	PCG	2	ycf4	PCG	3
ndhB-rps7	IGS	1	rps11	PCG	2	16SrRNA-trnL^{GAU}	IGS	3
ndhE-ndhG	IGS	1	rps15	PCG	2	5S-trnR^{ACG}	IGS	3
ndhH-rps15	IGS	1	rps16	PCG	2	accD-psaI	IGS	3
ndhK-ndhC	IGS	1	rps2	PCG	2	cemA-petA	IGS	3
petA-psbJ	IGS	1	rps3	PCG	2	ndhC-trnV^{UAC}	IGS	3
petB-petD	IGS	1	4.5S-5S	IGS	2	psbC-trnS^{UGA}	IGS	3
petG-trnW^{CAA}	IGS	1	atpB-rbcL	IGS	2	psbJ-psbL	IGS	3
psaA-ycf3	IGS	1	atpI-rps2	IGS	2	rpoB-trnC^{GCA}	IGS	3
psaC-ndhE	IGS	1	clpP-psbB	IGS	2	rpoC1-rpoB	IGS	3
psaJ-rps18 incl rpl33	IGS	1	ndhD-psaC	IGS	2	rps4-trnT^{UGU}	IGS	3
psbA-trnK	IGS	1	ndhF-rpl32	IGS	2	rps8-rpl14	IGS	3
psbE-petL	IGS	1	ndhG-ndhI	IGS	2	trnC^{GCA}-petN	IGS	3
psbI-trnS^{GCU}	IGS	1	ndhI-ndhA	IGS	2	rps12	intron	3
psbK-psbI	IGS	1	ndhJ-ndhK	IGS	2	trnI^{GAU}	intron	3
psbL-psbF	IGS	1	petD-rpoA	IGS	2	trnL	intron	3
psbZ-trnG^{GCC}	IGS	1	petL-petG	IGS	2	rRNAs_4	rRNA	3
rpl14-rpl16	IGS	1	petN-psbM	IGS	2	petG	PCG	4
rpl22-rps19	IGS	1	psaI-ycf4	IGS	2	23S-4.5S	IGS	4
rpl2-rpl23	IGS	1	psbB-psbT	IGS	2	psaI	PCG	5
rpoC2-rpoC1	IGS	1	psbH-petB	IGS	2	psbL	PCG	5
rps12-clpP exon3	IGS	1	psbM-trnD^{GUC}	IGS	2	atpA-atpF	IGS	5
rps15-ycf1	IGS	1	psbN-psbH	IGS	2	psbF	PCG	6
rps18-rpl20	IGS	1	psbT-psbN	IGS	2	rpl36	PCG	7
rps19-rpl2	IGS	1	rbcL-accD	IGS	2	rpoA-rps11	IGS	8
trnF^{GAA}-ndhJ	IGS	1	rpl16-rps3	IGS	2	ndhB	intron	9
trnfM^{CAU}-rps14	IGS	1	rpl20-rps12	IGS	2			
trnI^{CAU}-ycf2	IGS	1	rpl23-trnI^{CAU}	IGS	2			
